# The impact of developmental feedback on international students’ learning engagement: a mixed-methods study based on self-determination theory

**DOI:** 10.3389/fpsyg.2026.1858248

**Published:** 2026-05-22

**Authors:** Li Cai, Huaicheng Wu

**Affiliations:** 1 College of Foreign Languages, Shanghai Ocean University, Shanghai, China; 2 School of Chinese International Education, Shanghai University of International Business and Economics, Shanghai, China

**Keywords:** basic psychological needs, cross-cultural education, developmental feedback, learning engagement, mixed-methods design, self-determination theory

## Abstract

**Introduction:**

The rapid internationalization of higher education underscores the critical role of instructional support in facilitating international students’ academic integration. However, the underlying mechanisms through which teacher feedback strategies influence these students’ learning engagement in cross-cultural contexts warrant further investigation.

**Methods:**

Grounded in Self-Determination Theory (SDT), this study employed an explanatory sequential mixed-methods design within a Chinese Culture course. In the quantitative phase, 53 international students from two intact classes were assigned to either a developmental feedback condition (experimental group) or a traditional evaluative feedback condition (control group). This phase examined the differential effects of feedback strategies on learning engagement and the mediating role of basic psychological need satisfaction. Subsequently, in-depth interviews (*N* = 8) were conducted to unpack how students’ psychological needs were awakened and to identify the cognitive or environmental factors hindering the translation of psychological satisfaction into robust learning engagement.

**Results:**

Quantitatively, while developmental feedback significantly enhanced students’ psychological need satisfaction (autonomy and relatedness), it did not translate into increased learning engagement, indicating a disconnection between motivational satisfaction and behavioral investment. Qualitative findings illuminated the barriers to this translation, identifying cross-cultural cognitive overload, the “emotional comfort zone” effect, and the displacement of engagement into implicit forms. Furthermore, the analysis revealed that developmental feedback often functioned as a direct “task checklist” bypassing psychological mediation, while perceived over-feedback could trigger resistance.

**Discussion:**

The study challenges the linear assumption of Self-Determination Theory in cross-cultural contexts and provides nuanced insights for designing effective feedback in internationalized education.

## 1 Introduction

With the deepening of internationalization in higher education, the academic integration of international students within cross-cultural contexts has emerged as a core concern for universities worldwide. In this process, student engagement—defined as a persistent, pervasive, and positive affective-cognitive state—is manifested through high levels of energy and resilience, strong identification with the meaning of learning, and total immersion in academic pursuits (Fredricks et al., 2004). For international students navigating heterogeneous cultural environments, learning engagement is influenced not only by individual factors but is also profoundly reliant on external instructional support (Rienties et al., 2012). Among these support mechanisms, teacher feedback constitutes a pivotal element of high-frequency teacher-student interactions. However, traditional feedback is often perceived merely as a “summative evaluation” or a unidirectional “information correction,” which struggles to effectively motivate students undergoing cross-cultural adaptation (Evans, 2013).

In recent years, an ongoing paradigm shift in educational assessment has brought developmental feedback into focus as a forward-looking instructional intervention strategy. Unlike traditional reward-and-punishment or purely informational feedback, the core characteristics of developmental feedback lie in its “feed-forward” nature and “scaffolding” capabilities (Carless, 2006). It transcends merely judging the correctness of current tasks; rather, it is dedicated to providing specific steps for improvement, adopting a negotiatory and egalitarian dialogic tone, and respecting students’ academic autonomy within a sustained, non-punitive environment (Shute, 2008; Zhou, 2003). Particularly in courses such as Chinese Culture, which entail high cognitive loads and profound cultural barriers, international students face the dual challenges of language decoding and cultural schema acquisition. In this context, developmental feedback provided by teachers transcends mere knowledge transmission; it serves as a crucial nexus for offering cross-cultural cognitive scaffolds, alleviating academic anxiety, and rebuilding learning confidence among international students.

In recent years, research on feedback in higher education has undergone a profound paradigm shift, transitioning from a traditional “one-way transmission paradigm” to a focus on “Student Feedback Literacy” (Carless and Boud, 2018; Winstone et al., 2017). Emerging empirical evidence and meta-analyses suggest that even high-quality instructor feedback remains “inert information” if it is decoupled from the learner’s cognitive uptake and proactive engagement (Henderson et al., 2019).

This challenge is particularly salient within Second Language Acquisition (SLA) and international education contexts. Hyland and Hyland (2019) highlight that international students, when processing feedback embedded with dense cultural metaphors, experience significantly higher “cognitive load” and “emotional schema conflict” compared to domestic students. These findings suggest that the efficacy models of feedback developed in Western indigenous contexts cannot be universally applied. Instead, it is imperative to incorporate the cross-cultural cognitive boundaries of international students into the theoretical framework of feedback effectiveness.

Despite widespread academic recognition of the importance of instructional feedback, the underlying mechanisms through which developmental feedback translates into authentic learning engagement for international students require further exploration. First, from a theoretical perspective, existing research predominantly assumes a direct linear relationship between “feedback reception” and “behavioral change,” often overlooking the fact that feedback must trigger individuals’ internal psychological mechanisms to be effective (Molloy and Boud, 2013). Self-Determination Theory (SDT) posits that external environments can only be internalized into high-quality behavioral engagement if they satisfy three basic psychological needs: autonomy, competence, and relatedness (Deci and Ryan, 2000). However, facing a high-pressure, real-world academic ecology, once international students’ psychological needs are awakened, can they inevitably and smoothly overcome culture barriers and time conflicts to translate this satisfaction into visible learning engagement? Is there a possibility of “decoupling” in this “motivation-behavior” translation pathway? Current research has yet to thoroughly investigate this aspect.

Second, regarding research populations and methodologies, existing studies on feedback efficacy have predominantly focused on domestic student cohorts, with scarce exploration of how international students heterogeneously interpret feedback signals amidst complex cross-cultural challenges (Skyrme, 2007). Compared to domestic students, international students face far more severe cross-cultural adaptation pressures, language decoding barriers, and marginal identity dilemmas. Such cultural incongruity not only increases cognitive load but may also distort their perception of teachers’ feedback intentions. For example, they might misinterpret supportive developmental feedback as a questioning of their competence, or experience severe distortion of feedback signals during the decoding process due to culture barriers, thereby amplifying psychological defense mechanisms. Furthermore, traditional research has overly relied on single quantitative scale testing. While this method can measure static correlations between variables, quantitative data often fail to capture the deep emotional experiences behind international students’ knowing-doing disconnection when assumed pathways fail, nor can it explain counterintuitive phenomena such as intervention resistance potentially triggered by excessive feedback (Creswell and Plano Clark, 2018; Kahu, 2013).

In light of these gaps, this study targets international students in a Chinese Culture course and adopts an explanatory sequential mixed-methods design grounded in SDT. Initially, a quantitative survey is employed to test the hypothesized pathways among developmental feedback, basic psychological need satisfaction, and learning engagement. Subsequently, through in-depth qualitative interviews, this study endeavors to deconstruct the complex generative mechanisms hidden behind the quantitative model. This research aims not only to reveal how developmental feedback awakens students’ psychological satisfaction but also to explore what implicit cognitive and environmental barriers exist in authentic cross-cultural learning experiences, or whether there are shortcuts that bypass motivation. The findings are expected to provide theoretical explanations and practical guidance for optimizing teacher feedback strategies within the context of internationalized education. Building upon this foundation, the present study aims to investigate the effects of developmental feedback on international students’ academic engagement and the underlying psychological mechanisms. Employing an explanatory sequential mixed-methods design, the study first conducts hypothesis testing through a quantitative phase (RQs 1–3), followed by a qualitative phase (RQs 4–5) to provide a nuanced interpretation of the quantitative findings. The study addresses the following five research questions:

RQ1: Compared to the control group receiving traditional evaluative feedback, does the experimental group receiving the developmental feedback intervention achieve a significant enhancement in overall learning engagement?

RQ2: Following the developmental feedback intervention, do students in the experimental group exhibit significant positive changes in the three independent psychological dimensions of autonomy, competence, and relatedness needs?

RQ3: Do these three basic psychological needs significantly mediate the relationship between developmental feedback and academic engagement?

RQ4: Which features of developmental feedback effectively activate international students’ psychological needs in cross-cultural contexts?

RQ5: What latent barriers impede the translation of psychological satisfaction into engagement, and what alternative mechanisms facilitate direct engagement gains?

## 2 Literature review

### 2.1 International student engagement within the context of higher education internationalization

Student engagement is widely recognized as a critical indicator for predicting the quality of higher education, academic achievement, retention rates, and personal development (Kuh, 2001, 2009; Pascarella and Terenzini, 2005; Tinto, 2012). Within classical measurement frameworks, Schaufeli et al. (2002) defined engagement as a positive, fulfilling, and work-related state of mind characterized by three core dimensions: vigor, dedication, and absorption. Subsequent research has expanded this into a multidimensional construct encompassing behavioral (e.g., attendance, time spent on task), affective (e.g., sense of belonging and value alignment), and cognitive (e.g., use of deep learning strategies) components (Fredricks et al., 2004).

However, in the context of globalized higher education, the connotation and generative mechanisms of international student engagement exhibit high levels of specificity and situational contingency. Engagement serves not only as a metric for academic performance but also as a vital manifestation of cross-cultural adaptation and socio-psychological integration (Zhao et al., 2005). Rienties and Tempelaar (2013) noted that when studying in a host country, international students frequently encounter multifaceted challenges, including culture shock, and a lack of local social support. These exogenous stressors often result in initial levels of engagement—particularly explicit academic behaviors—that are significantly lower than those of their domestic peers.

This depletion of engagement is especially pronounced in courses with high indigenous cognitive attributes, such as Chinese Culture. International students must navigate unfamiliar academic concepts while simultaneously surmounting substantial cultural schema barriers. In such instances, the dual pressures of linguistic load and cognitive overload can trigger academic anxiety, which in turn inhibits deep cognitive engagement within traditional classroom environments (Andrade, 2006). Moreover, when faced with demanding multidisciplinary workloads, international students may engage in a zero-sum game of time and energy allocation between core major courses and general cultural electives, often resulting in strategically low levels of explicit engagement (Kahu, 2013).

Existing literature on international student engagement has largely focused on stable individual traits, such as self-efficacy and achievement motivation, or has relied on macro-level measurements of campus climate. These studies tend to overlook the micro-instructional environment—specifically, how concrete formative interventions by teachers serve as direct drivers of situational engagement (Zepke and Leach, 2010). Theoretically, according to SDT, external support should foster sustained deep engagement if it effectively satisfies students’ needs for autonomy, competence, and relatedness. However, whether this idealized pathway remains unobstructed in cross-cultural instructional settings requires empirical validation. Furthermore, traditional scales often restrict engagement to explicit indicators, such as the duration of assignment revisions or frequency of classroom participation, while neglecting the displacement of engagement forms—such as spontaneous cultural exploration or informal peer discussions (Kahu and Nelson, 2018). For experiential and internalized courses like Chinese Culture, these implicit forms of engagement may reflect a student’s degree of integration more accurately than explicit metrics.

Consequently, merely assessing individual motivation is insufficient to resolve the crisis of engagement among international students. There is a critical need to investigate which micro-instructional interventions—possessing both scaffolding properties and affective security (such as developmental feedback)—can effectively penetrate linguistic and cognitive barriers to activate authentic engagement. Addressing this theoretical blind spot is essential not only for advancing academic discourse but also for informing cross-cultural educational practice.

### 2.2 Teacher developmental feedback strategies

Serving as a critical nexus of instructional interaction, teacher feedback extends beyond mere pedagogical evaluation to function as a primary catalyst for students’ cognitive development. Classical models define feedback as information intended to “reduce discrepancies between current understandings and performance and a goal” (Hattie and Timperley, 2007). However, influenced by the evolution of constructivist and sociocultural learning theories, the conceptualization of feedback has undergone a significant paradigm shift. The focus has transitioned from traditional, unidirectional information transmission and retrospective summative evaluation toward a bidirectional, dialogic process and forward-looking intervention (Carless et al., 2011).

Rooted in this theoretical evolution, Teacher Developmental Feedback Strategies (TDFS) have emerged as a vital pedagogical tool. Transcending traditional error-correction, these strategies are defined as systematic interventions wherein educators intentionally provide scaffolded, negotiated, and forward-looking information tailored to specific academic contexts. This approach aims to foster students’ long-term learning and self-regulatory capacities, which is particularly crucial for international students navigating cross-cultural academic adaptation (Boud and Molloy, 2013; Carless, 2006). The efficacy of such feedback is not dictated solely by its timing or frequency; rather, it is profoundly contingent upon how its characteristics are perceived by the learners. Building upon Narciss (2008) functional dimensions, the current study conceptualizes developmental feedback across three highly complementary core dimensions:

Specific cognitive scaffolding: Moving beyond abstract critiques, this dimension constructs a cognitive scaffold by offering detailed, actionable improvement steps and resource pathways. Such high specificity reduces cognitive load, clarifies action pathways, and subsequently catalyzes deep learning (Kluger and DeNisi, 1996), thereby satisfying students’ psychological need for competence.

Positive affective valence: By employing genuine encouragement, affirmation, and a non-judgmental tone, educators convey psychological cues that students are recognized and accepted. In cross-cultural environments, feedback within this emotional dimension effectively mitigates inherent academic anxiety and frustration. It enhances international students’ self-efficacy and fosters a secure, trust-based teacher-student alliance (Steelman et al., 2004), ultimately fulfilling their need for relatedness.

Non-controlling negotiating discourse: Eschewing condescending or rigid directives, this approach utilizes autonomy-supportive language (e.g., “You have the option to...” or “I suggest considering...”). By emphasizing academic ownership, this narrative style protects intrinsic motivation and encourages students to autonomously initiate self-regulated learning within an atmosphere of mutual respect rather than coercion (Deci and Ryan, 2000), thereby supporting their need for autonomy.

Regarding the behavioral impact of these strategies, existing literature broadly supports the notion that high-quality formative feedback significantly predicts students’ academic performance and engagement levels. For instance, Jang et al. (2010) explicitly identify supportive instructional behaviors, including constructive feedback, as critical antecedents to student engagement. However, the current empirical landscape reveals two notable theoretical gaps: (1) Applicability of the Translation Model: While scholars hypothesize that high-quality feedback drives sustained engagement by satisfying basic psychological needs, it remains unclear whether this motivation-behavior translation model—originally established within domestic student populations—translates effectively to international students, who frequently contend with culture barriers and severe time constraints. (2) The Risk of Cognitive Overload: Prevailing studies frequently operate on the assumption that increased feedback detail inherently yields better outcomes. Yet, in high-pressure, cross-cultural academic ecosystems, there is a risk that excessively detailed “specific cognitive scaffolding” might overwhelm the cognitive processing capacity of international students. This potential cognitive overload could paradoxically inhibit their ability to translate feedback into actionable steps.

Feedback strategies influence international students’ learning engagement through the mediating role of basic psychological needs. By systematically uncovering the underlying translation mechanisms and potential boundary conditions, this research seeks to address these established gaps. Given the complexity of these micro-processes, addressing this theoretical objective necessitates a methodological departure from purely quantitative designs, demanding instead an in-depth qualitative exploration to accurately capture the nuanced realities of the international student experience.

## 3 Theoretical framework

### 3.1 Self-determination theory

This study adopts SDT as its overarching meta-theoretical framework. By contextualizing SDT through the integration of specific dimensions of teacher developmental feedback, this research constructs an integrated mediation model tailored for cross-cultural instructional settings. SDT posits that human beings are active organisms with an innate tendency toward psychological growth and integration; however, this process is fundamentally contingent upon the external environment’s support for three Basic Psychological Needs (BPN) (Deci and Ryan, 2000; Ryan and Deci, 2017): (1) Autonomy: The experience of being the origin of one’s own behavior, characterized by self-regulation and volitional freedom rather than external coercion. (2) Competence: The experience of efficacy and mastery when interacting with the environment, involving the confidence to exercise and extend one’s capabilities. (3) Relatedness: The experience of emotional connection and a sense of belonging within a community, marked by the feeling of being genuinely cared for and accepted by others. The satisfaction of these three needs is a prerequisite for the internalization of external regulations into self-endorsed values, which in turn stimulates high-quality behavioral engagement and psychological wellbeing (Vansteenkiste et al., 2020).

Notably, SDT has undergone substantial theoretical evolution within cross-cultural education and clinical psychology over the past decade. In their seminal reviews, Ryan and Deci (2020) and Vansteenkiste et al. (2020) emphasize that basic psychological needs are not limited to a unidirectional continuum of need satisfaction; rather, they encompass a discrete and independent dimension of need frustration.

Contemporary empirical research demonstrates that when external interventions—such as excessive academic demands or intrusive micromanagement—surpass an individual’s psychological capacity or cultural adaptation threshold, they do more than merely fail to provide satisfaction. Instead, such pressures can actively undermine autonomy and competence, subsequently inducing defensive avoidance and oppositional defiance. This theoretical progression provides a robust analytical lens for this study to explore the boundary conditions of developmental feedback and the underlying mechanisms of intervention resistance within the international student population. SDT is particularly suited for this study for several reasons. First, it explicitly delineates the internal psychological mechanisms—specifically BPN satisfaction—through which environmental factors (e.g., teacher feedback) influence behavioral outcomes (e.g., student engagement). Second, SDT’s emphasis on environmental affordances aligns with the conceptualization of developmental feedback as a proactive instructional provision. Finally, extensive literature has validated the efficacy of SDT in explaining second language acquisition and cross-cultural adaptation (Bureau et al., 2022; Hiver et al., 2021), providing a robust empirical foundation for the current inquiry.

### 3.2 Theoretical model construction: the integrated path of feedback, needs, and engagement

**Figure 1:**
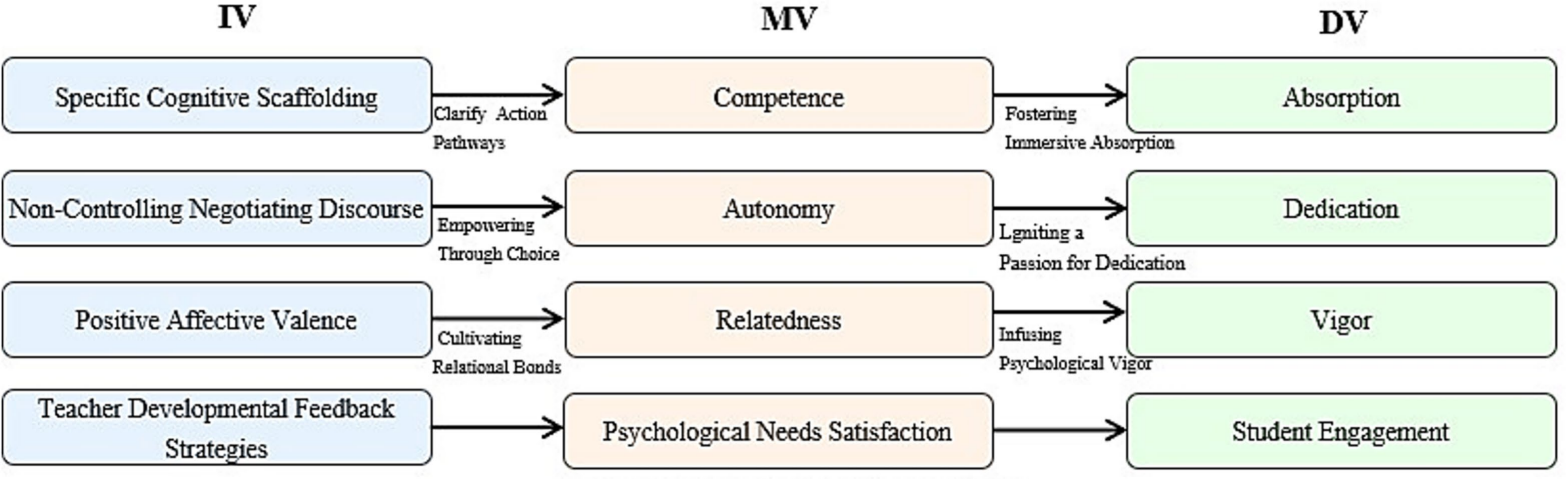
The integrated theoretical model construction: feedback, needs and engagement.

Grounded in the motivational internalization mechanisms of SDT, this study proposes an Integrated Mediation Model: *Teacher Developmental Feedback → Basic Psychological Needs Satisfaction → Student Engagement* (Figure 1). In this model, developmental feedback functions as a contextual affordance, while student engagement is viewed as the behavioral “emergence” resulting from the precise “nourishment” of intrinsic psychological needs. Within this logical chain, the three basic psychological needs serve as critical psychological hubs.

#### 3.2.1 Developmental feedback as contextual affordance

The study hypothesizes that specific dimensions of feedback correspond to the satisfaction of specific needs. Specific cognitive scaffolding directly addresses the need for competence by clarifying actionable pathways and reducing ambiguity. This aligns with the “New Feedback Paradigm” (Winstone and Carless, 2020), which views feedback as a catalyst for student self-regulation rather than mere information transmission.

In high-cognitive-load contexts, such as courses on “Chinese Culture,” such scaffolding reconstructs students’ self-efficacy (Hattie and Timperley, 2007). Furthermore, non-controlling negotiating discourse fulfills the need for autonomy by granting academic ownership and reducing perceived pressure, consistent with autonomy-supportive instructional perspectives (Reeve, 2009). Finally, positive affective valence nourishes the need for relatedness. The non-evaluative care and encouragement embedded in such feedback establish a psychological safe base, which is vital for international students facing the stressors of a foreign academic environment.

#### 3.2.2 Psychological needs satisfaction as the internal engine for engagement

The model further hypothesizes that BPN satisfaction acts as the core internal engine propelling students from passive compliance to deep engagement. When these needs are met, motivation undergoes a qualitative shift from external regulation (e.g., studying to avoid failure) to integrated regulation (e.g., studying due to a deep endorsement of learning values). Empirical evidence suggests that competence is a powerful predictor of the absorption dimension of engagement, allowing students to achieve a state of flow even during challenging tasks (Csikszentmihalyi, 1990). A sense of autonomy ignites dedication; when learning is perceived as a volitional choice, students develop emotional identification with the subject matter (Skinner et al., 2009). Lastly, relatedness provides the psychological resilience—or vigor—necessary to maintain participation despite the linguistic and cultural setbacks inherent in international education (Carmichael et al., 2017).

#### 3.2.3 Boundary conditions and the rationale for mixed-methods

While the classic SDT model provides a strong foundation, its application in complex cross-cultural ecologies may reveal certain boundary conditions. For instance, the linear path from needs satisfaction to engagement may be disrupted by factors such as cognitive overload. Paradoxically, over-feedback—even when intended as scaffolding—may be perceived as controlling pressure, thereby inhibiting autonomy. To address these potential theoretical limits and capture the nuanced micro-processes of feedback internalization, this study employs a mixed-methods design. This approach allows for qualitative data to illuminate the underlying mechanisms and causal logics behind these interactions, bridging the gap where purely quantitative models may lack explanatory depth.

### 3.3 A motivation–cognition dual-track conversion framework in cross-cultural contexts

Recognizing the potential boundary limitations of the classical SDT model in explaining high-stakes, cross-cultural academic ecosystems, this study seeks to extend beyond motivational arousal. By integrating SDT with an information-processing perspective—specifically Cognitive Load Theory (CLT; Sweller, 1988)—we propose a unified “motivation-cognition dual-track conversion” framework.

Within this integrated framework, SDT and CLT are not viewed as disparate theories but as a synergistic mechanism of “will” and “capacity.” SDT elucidates *why* international students are willing to engage (the motivational engine), under the implicit premise that they possess sufficient cognitive resources. Conversely, CLT defines the prerequisite boundary conditions for this motivational conversion. When international students encounter the dual challenges of linguistic decoding and cultural schema deficits, they are prone to cognitive overload, which subsequently disrupts the “motivation-to-behavior” pathway predicted by SDT.

Consequently, this framework posits that effective developmental feedback must serve a dual function: (1) Affective Support: Utilizing dialogic and negotiated discourse to trigger the satisfaction of SDT-related psychological needs. (2) Cognitive Offloading: Providing structural scaffolding through specific feed-forward strategies to minimize extraneous cognitive load.

This unified framework provides a coherent theoretical lens for both testing quantitative hypotheses and deconstructing the qualitative mechanisms underlying potential pathway disconnects.

## 4 Methodology

### 4.1 Study design

To address the research questions comprehensively, this study adopts an explanatory sequential mixed-methods design (Creswell and Plano Clark, 2018). Following a quantitative-dominant (QUAN–qual) approach, the study leverages qualitative data to elucidate the underlying micro-mechanisms and latent causalities that inform the quantitative findings.

The first phase utilized a quasi-experimental, longitudinal design to address RQ1, RQ2, and RQ3. Two intact, parallel classes were designated as the experimental and control groups to investigate the longitudinal effects of a developmental feedback intervention via multi-wave survey administration.

The second phase employed a qualitative approach utilizing semi-structured interviews to address RQ4. Guided by the preliminary quantitative findings, extreme case sampling was utilized to select representative participants from the experimental group. The qualitative data aimed to triangulate the quantitative findings and deeply delineate the underlying psychological mechanisms—specifically, how students subjectively perceived the intervention and how these perceptions translated into intrinsic psychological drives.

### 4.2 Participants

The study was conducted within a compulsory general education course, at a university on the eastern coast of China. Using convenience sampling, an initial cohort of 56 international students from two naturally occurring, intact classes was recruited. To ensure homogeneity in cultural backgrounds and psychological baselines (Ryan and Reid, 2016), three ethnic Chinese international students (two from the experimental group and one from the control group) were excluded *a priori* from the analysis. This resulted in a final valid sample of 53 participants (*N* = 53).

This exclusion is grounded in established empirical evidence from SLA and motivational psychology. Within the specific context of a “Chinese Culture” course, these individuals are classified as heritage learners. Research indicates that heritage learners possess fundamentally different prior cultural capital and motivational structures compared to non-heritage international students (Valdés, 2005). Within the framework of SDT, Comanaru and Noels (2009) demonstrated that due to early socialization and familial environments, heritage learners often exhibit significantly higher baseline satisfaction in relatedness and competence when engaging with their ancestral culture.

Including participants with such high cultural proximity in this quasi-experimental study (*N* = 56) would likely induce a ceiling effect and introduce confounding variables, thereby distorting the net effect of the developmental feedback intervention. Consequently, the exclusion of heritage learners was a necessary control procedure to mitigate selection bias and safeguard the internal validity and homogeneity of the sample.

Class A served as the experimental group (*n* = 27) and Class B as the control group (*n* = 26). The participants represented 10 nationalities, including Russia, Thailand, South Korea, Indonesia, and Kazakhstan. The mean age was 22.0 years (*SD* = 1.800) for the experimental group and 22.1 years (*SD* = 1.900) for the control group, revealing no significant between-group difference (*t* = −0.196, *p* > 0.050). Both groups were predominantly composed of STEM majors (e.g., aquaculture, engineering, information technology), supplemented by a minority of social science majors. All participants had resided in China for 0.5 to 1 year and had ensured sufficient linguistic capacity to comprehend both the instructional content and the survey instruments. Chi-square and independent samples *t*-tests confirmed no significant baseline differences between the groups regarding age, gender composition, or academic major (*p* > 0.050; Table 1).

**Table 1:** Demographic characteristics of the experimental and control groups (Fall 2024).

Group	Classes (*n*)	Initial sample size (*N*)	Excluded participants (*n*)	Valid participants (*n*)	Age (*M ± SD*)	Gender ratio (M: F)	Major ratio (STEM: Social Sciences)
EG	1 (Class A)	29	2	27	22.000 ± 1.800*	12:13	23:2
CG	1 (Class B)	27	1	26	22.100 ± 1.900*	14:14	25:3
*T*	--	--	--	--	*t* = −0.196	χ^2^ = 0.021	χ^2^ = 0.114
*p*	--	--	--	--	*p* > 0.050	*p* = 0.885	*p* = 0.736

*p < 0.05.

To mitigate potential threats to internal validity inherent in the quasi-experimental design, several confounding variables were rigorously controlled.

First, to account for baseline prior knowledge, both groups completed a standardized academic pre-test developed in accordance with the course content. An independent samples *t*-test revealed no significant difference between the experimental group (*M* = 19.100, *SD* = 6.190) and the control group (*M* = 19.260, *SD* = 6.120), (*t* = −0.130, *p* = 0.851). These results indicate that the two groups were equivalent in their baseline academic proficiency.

Second, to neutralize systematic variance arising from instructional delivery, both groups were taught by the same instructor. Furthermore, the two groups utilized consistent course materials and assessment criteria. This standardization effectively shielded the study from potential biases related to individual teaching styles or content disparities. By doing so, the study maintained high ecological validity while concurrently safeguarding the internal validity of the quasi-experimental intervention.

To ensure sufficient statistical sensitivity and mitigate potential bias, rigorous control measures were implemented throughout the experimental design and execution phases.

First, regarding the adequacy of the sample size, an *a priori* power analysis was conducted using G*Power 3.1 (Faul et al., 2007) prior to the intervention. For the core statistical procedures—specifically the univariate ANCOVA and the 2 × 5 mixed Repeated Measures ANOVA—the minimum sample size required to detect a medium effect size (*f* = 0.25) with a significance level of *ɑ* = 0.05 and a power level of 1-*β* = 0.80 was determined to be approximately 45. The final sample of *N* = 53 exceeded this theoretical threshold. Furthermore, a *post-hoc* power analysis revealed that, due to the large observed effect sizes yielded by the intervention (e.g., partial eta squared $\eta_{p}^{2}$ *=* 0.872 in the ANCOVA), the actual statistical power exceeded 0.95. This demonstrates that the current sample size was robust and sufficient for detecting significant differences between groups.

Second, regarding bias control and internal validity, the study addressed the inherent risks of non-random assignment associated with the quasi-experimental design through multiple controls: (1) Equivalence Testing: Homogeneity tests (Tables 1, 2) confirmed that the two intact classes were statistically equivalent across key demographic variables and academic baselines. (2) Isolation of Confounding Variables: Course content and materials are presented in both Chinese and English to eliminate interference from language comprehension barriers on intervention outcomes. (3) Control for Teacher and Environmental Effects: To minimize extraneous variance, both the experimental and control groups were taught by the same trained instructor. Additionally, instructional objectives, learning materials, task difficulty, and assessment systems were kept identical across both conditions, with “feedback type” as the sole manipulated variable.

**Table 2:** Comparison of pre-test mean scores and standard deviations between the EG and CG.

EG pre- test		CG pre- test		Paired *t*-test results	
Mean	SD	Mean	SD	*t*	*p*
19.100	6.190	19.260	6.120	−0.130	0.851

*p < 0.05.

Through these methodological safeguards, the study maintained high ecological validity within an authentic classroom setting while maximizing internal validity by effectively mitigating selection bias and confounding factors.

### 4.3 Procedure and intervention

The quasi-experiment spanned 16 weeks, comprising two instructional hours per week. To strictly control for extraneous variables, both classes were taught by the same experienced instructor and utilized identical textbooks, micro-lecture videos, and assignments. The sole manipulated independent variable was the teacher feedback strategy.

Control group: Participants received traditional, verificative evaluations. The instructor primarily provided dichotomous judgments or generalized praise, offering minimal specific improvement strategies or personalized emotional support.

Experimental group: Participants received developmental feedback specifically designed to nourish basic psychological needs. This multidimensional intervention comprised: (1) Specific cognitive scaffolding (supporting *competence*): Providing clear task criteria and actionable improvement strategies. (2) Non-controlling negotiating discourse (supporting *autonomy*): Utilizing suggestive rather than imperative language to validate students’ unique insights. (3) Positive affective valence (supporting *relatedness*): Employing encouraging vocabulary, focusing on individual progress, and using inclusive pronouns to foster connection.

Following the completion of the 16-week quantitative phase, extreme case sampling (a purposive sampling technique) was employed to select eight representative students from the experimental group for the qualitative interview phase.

### 4.4 Measurement instruments

The quantitative instruments comprised three self-report scales measuring Perceived Teacher Feedback, Basic Psychological Needs, and Student Engagement. Data were collected at five distinct time points: baseline (W0), and the end of weeks 4 (W4), 8 (W8), 12 (W12), and 16 (W16). All items were evaluated on a 7-point Likert scale ranging from 1 (*Strongly Disagree/Never*) to 7 (*Strongly Agree/Always*). Given the demographic makeup of the sample, bilingual (Chinese-English) questionnaires were administered, developed through a rigorous back-translation procedure to guarantee semantic equivalence.

To facilitate longitudinal data matching while strictly preserving confidentiality, participants provided their student IDs, with explicit written assurances that all data would be anonymized using alphanumeric codes (e.g., S1, S2) for analysis and publication. The W16 survey also included an opt-in prompt for the subsequent interview phase.

Perceived Teacher Feedback Scale: Adapted from Koka and Hein (2003) and contextually revised for this study. The dimensions were mapped to the intervention: “informational/instructional feedback” to specific cognitive scaffolding; “controlling/non-controlling feedback” (reverse-scored) to non-controlling negotiating discourse; and “positive/general feedback” to positive affective valence. The scale demonstrated robust internal consistency (Cronbach’s *α* = 0.84).

Basic psychological needs scale (BPNS): Developed by Gagné (2003) and revised by Tian et al. (2014), this 15-item scale assesses autonomy, competence, and relatedness. In the current study, the subscales exhibited satisfactory reliability (Cronbach’s α = 0.87, 0.87, and 0.70, respectively).

Utrecht work engagement scale-student (UWES-S): Adapted from Schaufeli et al. (2002) and contextually tailored, this 9-item scale measures engagement across three dimensions: vigor, dedication, and absorption. The revised scale demonstrated strong reliability (Cronbach’s α = 0.82).

### 4.5 Qualitative interview design

To address RQ4, eight students from the experimental group, selected via extreme case sampling to capture maximum variation in intervention response, participated in semi-structured interviews. The interview protocol consisted of four thematic modules (Appendix 1). Interviews lasted 20–30 min, were audio-recorded with consent, and were subsequently transcribed verbatim for analysis.

### 4.6 Data analysis

In accordance with the explanatory sequential design, data analysis was executed in two distinct phases: quantitative hypothesis testing followed by qualitative mechanism exploration.

1. Quantitative analysis

Data processing and statistical analyses were conducted using SPSS 26.0. Initial analyses included descriptive statistics and Pearson correlations to evaluate the baseline associations among developmental feedback, basic psychological needs, and student engagement. Subsequently, given the quasi-experimental nature of this study and the sample size (*N* = 53), several methodological safeguards were employed to ensure statistical robustness and mitigate the risks of Type I and Type II errors.

Regarding the 2 × 5 mixed-design ANOVA, to prevent inflated Type I errors resulting from potential violations of the sphericity assumption in small samples, a conservative Greenhouse–Geisser (GG) correction was applied to all within-subjects effects. Furthermore, a post-hoc power analysis indicated that, due to the substantial observed effect sizes (e.g., $\eta_{p}^{2}$ = 0.250), the study maintained an actual statistical power exceeding 0.95, ensuring the stability and reliability of the longitudinal comparisons.

For the parallel mediation analysis, traditional Structural Equation Modeling (SEM) was deemed inappropriate due to its stringent requirements for large samples (*N* > 200) and multivariate normality. Consequently, we utilized the PROCESS macro (Model 4; Hayes, 2018) based on observed-variable path analysis. This approach employs 5,000 bias-corrected bootstrap resamples to estimate 95% confidence intervals for indirect effects. Methodological research has confirmed that for intervention studies with small-to-moderate samples, bootstrapping is the gold standard for maintaining statistical power and enhancing parameter robustness (Preacher and Hayes, 2008). Additionally, multiple imputation techniques were applied to address potential data attrition inherent in the longitudinal design, ensuring unbiased parameter estimation.

1. Qualitative analysis

A hybrid deductive-inductive thematic analysis approach (Fereday and Muir-Cochrane, 2006) was employed. Transcripts were imported into NVivo 12 and subjected to a rigorous three-level coding process: open, axial, and selective coding.

To establish the trustworthiness and rigor of the qualitative findings (Lincoln and Guba, 1985), two primary strategies were implemented: (1) Investigator Triangulation: Two independent researchers with backgrounds in educational psychology conducted “back-to-back” coding. The initial inter-coder reliability (Cohen’s Kappa) was robust at 0.85. Any interpretive discrepancies were resolved through iterative re-evaluation of the raw corpus and consensus-building discussions. (2) Member Checking: Upon extraction of the core categories and learner typologies, preliminary findings were shared with selected interviewees (e.g., those exhibiting “Decoupled” or “Resistant” response patterns) to verify the authenticity and accuracy of the researchers’ interpretations regarding their psychological experiences.

### 4.7 Ethical considerations

The study protocol was approved by the Ethics Committee of the University. Prior to data collection, an information sheet detailing the study’s objectives and procedures was distributed to all participants. Written informed consent was obtained, explicitly acknowledging voluntary participation, the right to withdraw at any time without academic penalty, and the strict anonymization of all data for academic use.

## 5 Results

### 5.1 The effect of developmental feedback on student engagement (RQ1)

To determine whether the developmental feedback intervention (experimental group) significantly enhanced overall student engagement compared to traditional evaluative feedback (control group), a one-way analysis of covariance (ANCOVA) was conducted. The post-test learning engagement score served as the dependent variable, while the pre-test learning engagement score was entered as a covariate to control for baseline differences.

Preliminary assumption testing confirmed the homogeneity of regression slopes, as evidenced by a non-significant interaction between the group and the pre-test scores (*p* = 0.494), thereby validating the use of ANCOVA. The ANCOVA results (Table 3) indicated that, after partialling out the effect of the baseline pre-test scores, a statistically significant difference remained between the two feedback groups regarding post-test overall learning engagement, *F*(1, 50) *=* 340.179*, p <* 0.001, $\eta_{p}^{2}$ *=* 0.872. Specifically, as detailed in Table 4, the covariate-adjusted marginal mean for the experimental group (*M* = 46.780, *SE* = 0.560) was significantly higher than that of the control group (*M* = 33.190, *SE* = 0.470). This finding demonstrates that students receiving the developmental feedback intervention achieved a substantial enhancement in their overall learning engagement. The uniquely large effect size ( $\eta_{p}^{2}=0.872$ ) indicates that developmental feedback exerts a robust and practically meaningful positive effect on student engagement.

**Table 3:** Analysis of covariance (ANCOVA) summary for post-test learning engagement.

Source	*SS*	*df*	*MS*	*F*	*p*
Intercept	1157.221	1	1157.221	170.850	< 0.001**
Group	2304.134	1	2304.134	340.179	< 0.001**
Pre-test (Covariate)	24.040	1	24.040	3.549	0.065
Error	338.665	50	6.773		

*R*^2^ = 0.879. *SS* = sum of squares; *df* = degrees of freedom; *MS* = mean square. *p < 0.05, **p < 0.01.

**Table 4:** Estimated marginal means and standard errors for post-test learning engagement by group.

Goup	*M*	*SE*	*n*
EG	46.780	0.560	27
CG	33.190	0.470	26

### 5.2 Longitudinal effects on basic psychological needs (RQ2)

To investigate whether students in the experimental group exhibited significant positive changes across the three independent psychological dimensions (autonomy, competence, and relatedness) over time, a 2 (Group) × 5 (Time) mixed-design repeated-measures analysis of variance (ANOVA) was conducted.

Prior to the main analysis, Mauchly’s test of sphericity was performed for the within-subjects effects across all three dimensions. The results indicated that the assumption of sphericity was violated for autonomy (*W* = 0.408, *p* < 0.001), competence (*W* = 0.572, *p* = 0.001), and relatedness (*W* = 0.533, *p* < 0.001). Consequently, the Greenhouse–Geisser correction was uniformly applied to adjust the degrees of freedom and -values for all within-subjects effects. The comprehensive ANOVA results are presented in Table 5.

**Table 5:** Summary of mixed-design repeated measures ANOVA for basic psychological needs.

Dimension	Source of Variation	*df*	*F*	*p*	${?}_{p}^{2}$
Autonomy	Between-subjects (Group)	1	73.347	<0.001**	0.590
	Within-subjects (Time)†	3.072	105.509	<0.001**	0.674
	Interaction (Group × Time)†	3.072	28.588	<0.001**	0.359
Competence	Between-subjects (Group)	1	3.108	0.084	0.057
	Within-subjects (Time)†	3.184	100.403	<0.001**	0.663
	Interaction (Group × Time)†	3.184	11.410	<0.001**	0.183
Relatedness	Between-subjects (Group)	1	28.696	<0.001**	0.360
	Within-subjects (Time)†	3.251	75.326	<0.001**	0.596
	Interaction (Group × Time)†	3.251	33.264	<0.001**	0.395

^†^Results were adjusted using the Greenhouse–Geisser correction. **indicate *p* < 0.001 (exact value).

The empirical data revealed that the impact of developmental feedback on the three basic psychological needs was not a uniform, linear progression; rather, it exhibited highly differentiated, dynamic trajectories.

#### 5.2.1 Autonomy: immediate activation and sustained growth

**Figure 2:**
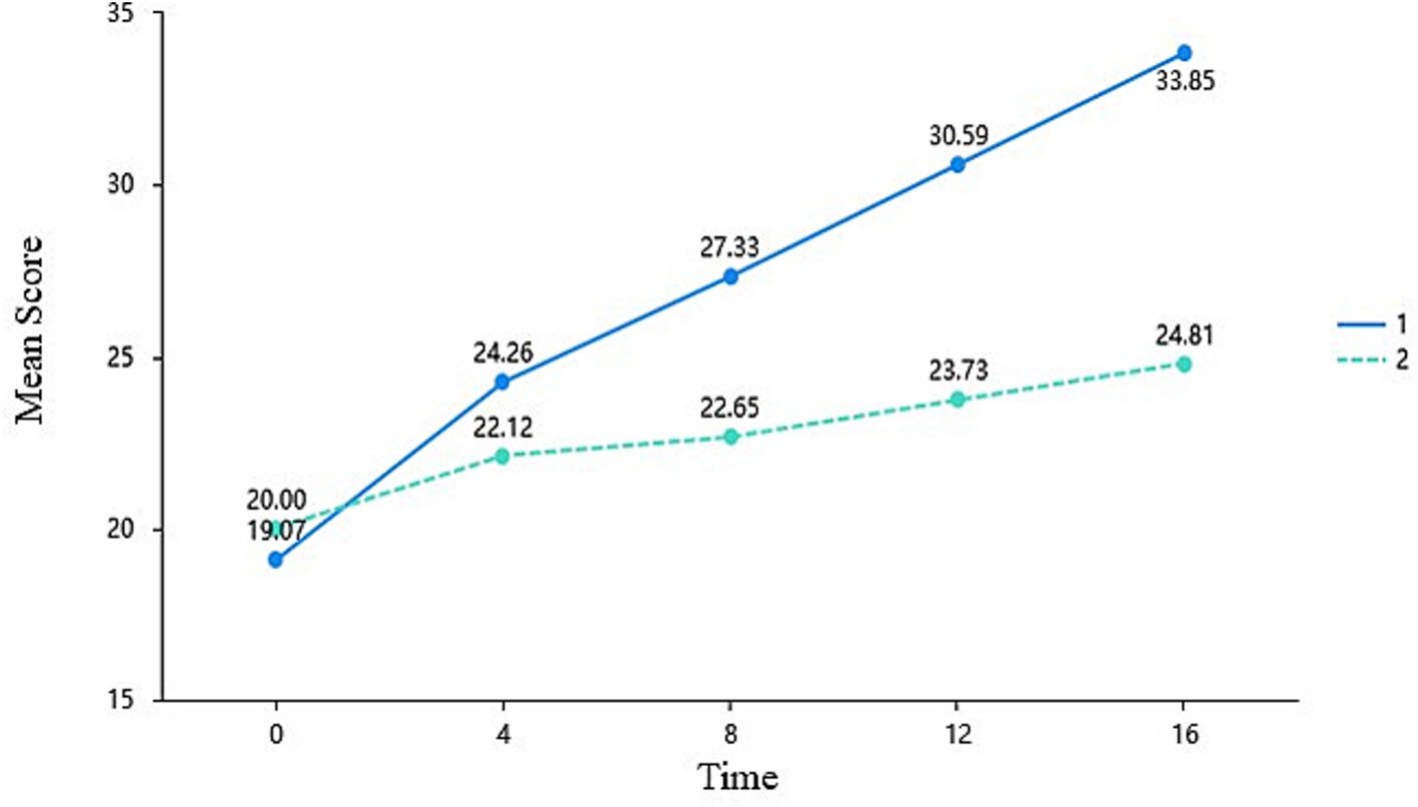
Mean differences in the need for autonomy across time and groups (EG = 1, CG = 2).

Developmental feedback activated students’ need for autonomy most rapidly and directly. Results revealed a highly significant main effect of Time (*F* = 105.509, *p* < 0.001) and Group (*F* = 73.347, *p* < 0.001). Crucially, the Time × Group interaction effect reached profound significance with a large effect size, *F* = 28.588, *p* < 0.001, ${?}_{p}^{2}$ = 0.359. Simple effects analysis further indicated that, while there was no significant difference between the two groups at baseline (T0, *p* = 0.265), the experimental group demonstrated significantly higher perceived autonomy than the control group beginning at Week 4 (*p* = 0.010). As illustrated in Figure 2, while the control group experienced a marginal natural increment of 4.81 points over the 16 weeks, the experimental group achieved a rapid increase of 14.78 points, underscoring the immediate efficacy of the intervention in facilitating psychological empowerment.

#### 5.2.2 Competence: initial adaptive fluctuation and subsequent leapfrog breakthrough

**Figure 3:**
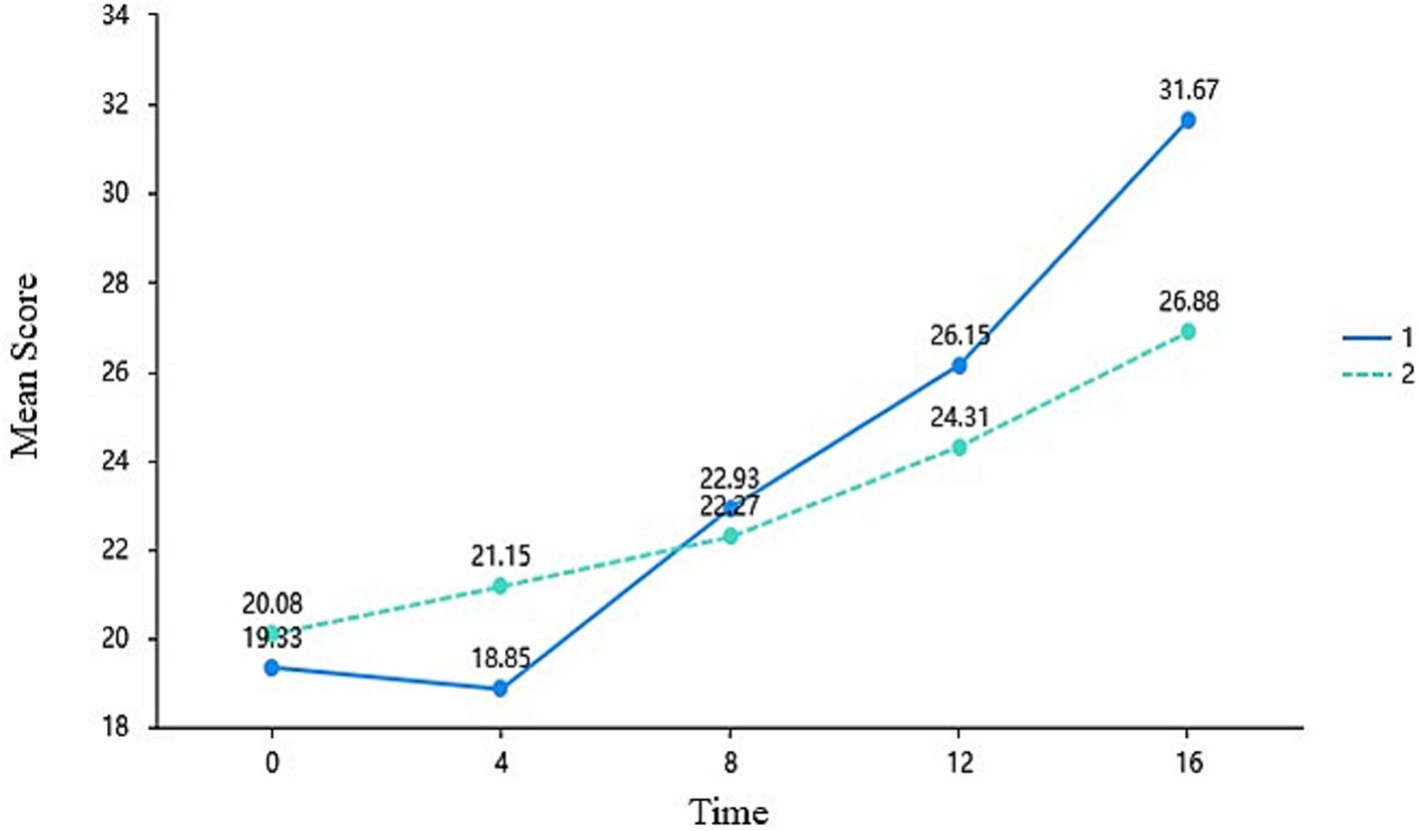
Mean differences in the need for competence across time and groups (EG = 1, CG = 2).

The temporal trajectory of competence satisfaction illustrates a distinct “cognitive load adaptation phase,” a hallmark characteristic of high-challenge interventions. Although the main effect of Time was highly significant (*F* = 100.403, *p* < 0.001), the main effect of Group was only marginally significant (*p* = 0.084). However, the Time × Group interaction effect was highly significant, *F* = 11.410, *p* < 0.001, $\eta_{p}^{2}$ = 0.183. Notably, Figure 3 illustrates that students’ competence in the experimental group did not initially increase at Week 4; instead, it experienced a slight downward oscillation (*M* = 18.850) compared to the baseline (*M* = 19.330). Following this initial cognitive adjustment period, the experimental group’s competence scores surged from Week 8 onwards. Their final score (*M* = 31.670) significantly surpassed that of the control group (*M* = 26.880), which relied purely on natural maturation over the course duration.

#### 5.2.3 Relatedness: prolonged latency and late-stage eruption

**Figure 4:**
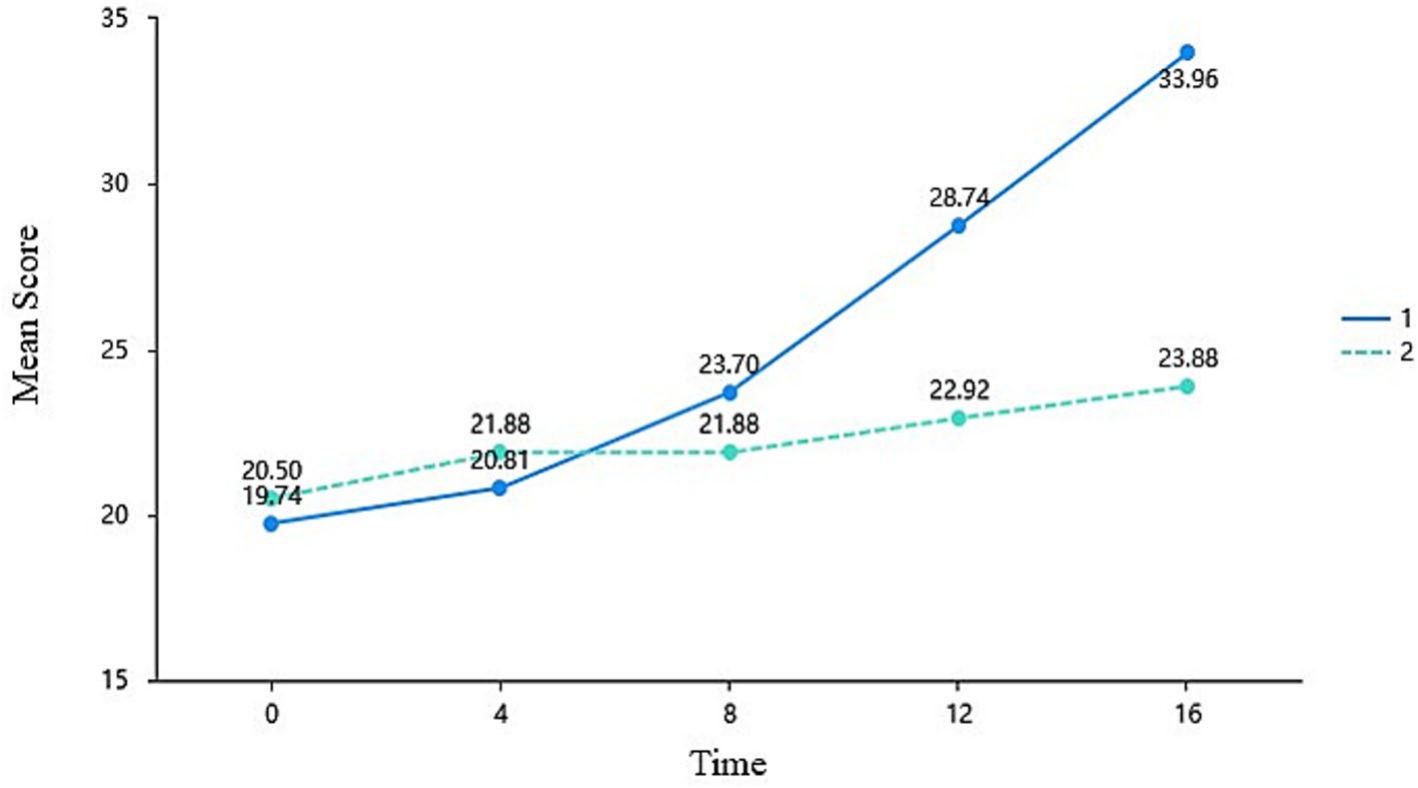
Mean differences in the need for relatedness across time and groups (EG = 1, CG = 2).

The reconstruction of relatedness proved to be the most protracted process; however, its cumulative intervention effect was the most profound. The Time × Group interaction effect was exceptionally significant, *F* = 33.264, *p* < 0.001, with its effect size ( $\eta_{p}^{2}$ = 0.395) ranking highest among the three psychological dimensions. Simple effects analysis indicated a latency period during the first 4 weeks of the intervention, with no significant between-group impact on teacher-student or peer relationships (*p* = 0.423 and *p* = 0.259, respectively). As shown in Figure 4, the divergence between the two groups emerged at Week 8 and widened substantially at Weeks 12 and 16. Over the 16-week period, the experimental group’s relatedness score increased by 14.22 points, contrasting sharply with a nominal increase of 3.38 points in the control group. This highlights the pivotal role of developmental feedback in cultivating a sustainable, deep-learning community over time.

### 5.3 Synthesis: asymmetrical growth and correlation analysis

Synthesizing the longitudinal data across these dimensions reveals a distinct pattern of *asymmetrical growth* as developmental feedback restructures students’ psychological micro-ecosystems.

First, regarding intervention intensity, developmental feedback stimulated the basic psychological needs with clear hierarchical disparities. The interaction effect sizes for relatedness ( $\eta_{p}^{2}\;$ = 0.395) and autonomy ( $\eta_{p}^{2}$ = 0.359) were approximately double that of competence ( $\eta_{p}^{2}$ = 0.183). This provides robust quantitative evidence for the application of SDT in educational contexts: compared to directly enhancing students’ efficacy judgments regarding skill mastery (competence), developmental feedback reaches much deeper into their emotional connection networks and perceived locus of learning control.

Second, regarding dynamic temporal evolution, the breakout points for the three dimensions were distinctly staggered. Autonomy was activated rapidly in the early stages (Week 4). Competence required a period of cognitive dissonance and challenge, breaking through in the middle stage (Week 8). Conversely, relatedness exhibited a “delayed onset” pattern, requiring a prolonged accumulation of bidirectional trust before delivering the most robust psychological empowerment in the later stages. This dynamic chain—*early awakening of motivation → oscillatory reconstruction of ability → deep binding of relationships*—elucidates how developmental feedback fosters enduring change within students’ psychological systems.

**Figure 5:**
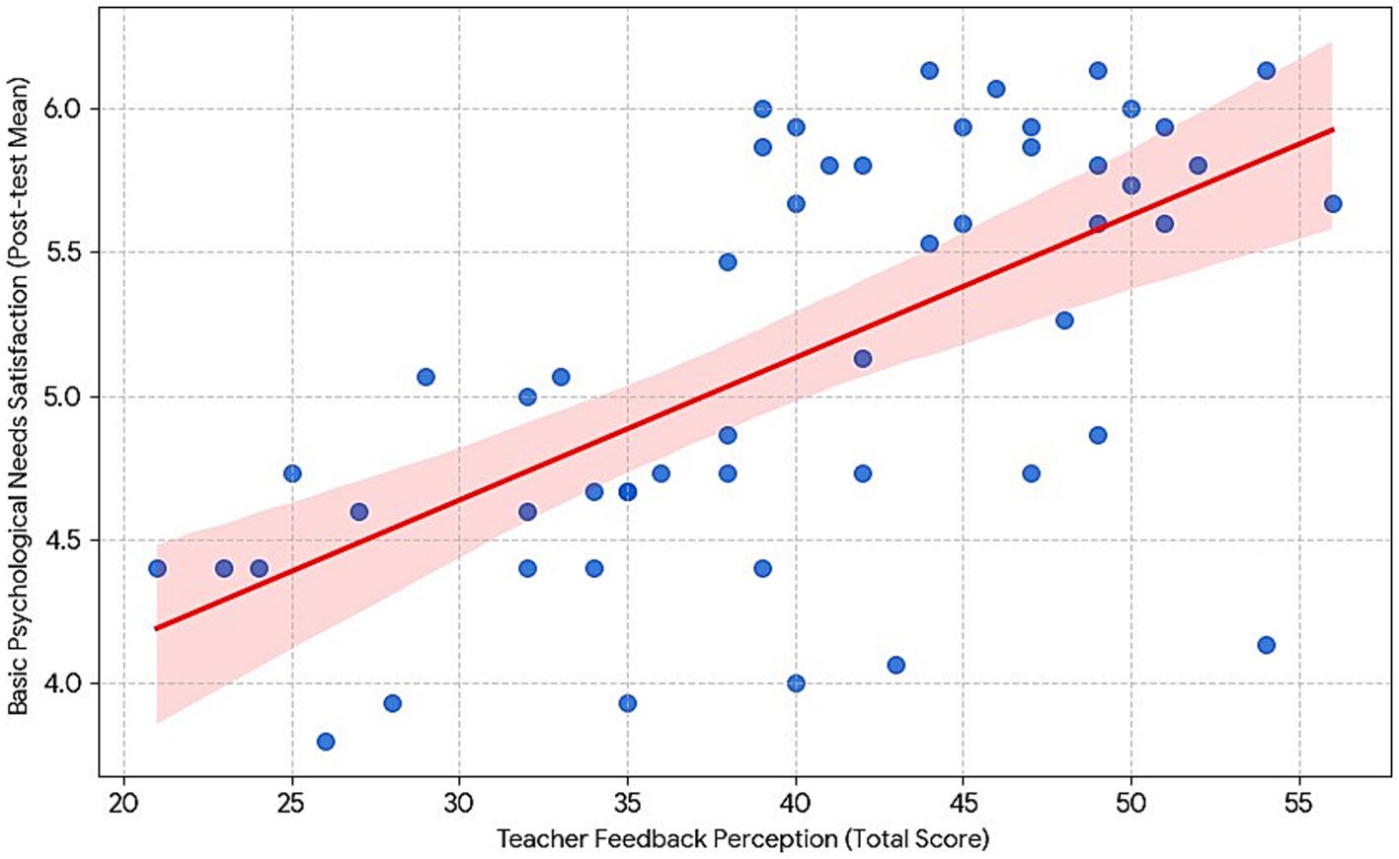
Correlation between teacher feedback and psychological needs.

Finally, as depicted by the scatterplot and corresponding statistical results (Figure 5), there was a significant positive correlation between perceived teacher feedback and basic psychological needs satisfaction (*r* = 0.618, *p* < 0.001). The regression line clearly illustrates this positive linear trend: the more supportive the feedback perceived by the students, the greater the satisfaction of their intrinsic psychological needs.

### 5.4 Mediation analysis (RQ3)

To address RQ3—whether the basic psychological needs for autonomy, competence, and relatedness significantly mediate the relationship between teacher developmental feedback and student learning engagement—a parallel mediation model was employed. The significance of the indirect effects was evaluated using a bias-corrected bootstrapping approach with 5,000 resamples. In this model, Group (experimental vs. control) served as the independent variable, and post-test overall learning engagement (W16) was designated as the dependent variable. The post-test scores of the three psychological needs (W16) were entered as parallel mediators, while their baseline levels (W0) were strictly controlled as covariates.

Path analysis results confirmed that teacher developmental feedback significantly predicted student learning engagement. The total effect of the feedback intervention (Group) on learning engagement was statistically significant (Total Effect = −13.291, *t* = −17.044, *p* < 0.001). The negative coefficient in this coding scheme indicates that the experimental group exhibited significantly higher levels of learning engagement compared to the control group. Upon introducing the three mediating variables into the model, the direct effect of Group on learning engagement remained highly significant (Direct Effect = −15.103, *t* = −6.705, *p* < 0.001). This initially suggests that the influence of developmental feedback on learning engagement operates predominantly through a direct pathway.

Further decomposition of the mediation pathways (Path *a*) revealed that the intervention significantly reshaped students’ psychological need satisfaction. Specifically, Group significantly predicted the need for autonomy (*B* = −8.999, *p* < 0.001), competence (*B* = −4.552, *p* < 0.001), and relatedness (*B* = −9.998, *p* < 0.001). However, after controlling for the effect of Group, the predictive power of the three psychological needs on learning engagement (Path *b*) failed to reach statistical significance. The individual impacts of autonomy (*B* = −0.038, *p* = 0.853), competence (*B* = −0.080, *p* = 0.662), and relatedness (*B* = −0.110, *p* = 0.447) on learning engagement were all non-significant (Table 6).

**Table 6:** Summary of regression analysis results for paths in the parallel mediation model.

Path category	Path	*B*	*SE*	*t*	*p*
Total and direct effects	Group → Learning engagement (Total Effect)	−13.291	0.780	−17.044	< 0.001***
	Group → Learning engagement (Direct effect)	−15.103	2.253	−6.705	< 0.001***
Paths (IV → Mediators)	Group → Autonomy	−8.999	0.582	−15.471	< 0.001***
	Group → Competence	−4.552	0.691	−6.590	< 0.001***
	Group → Relatedness	−9.998	0.842	−11.879	< 0.001***
Paths (Mediators → DV)	Autonomy → Learning engagement	−0.038	0.206	−0.186	0.853
	Competence→Learning engagement	−0.080	0.182	−0.440	0.662
	Relatedness→Learning engagement	−0.110	0.144	−0.766	0.447

The model controlled for the W0 baseline variable. IV = Independent Variable; DV = Dependent Variable. **p < 0.001.

Consequently, the bootstrap confidence intervals confirmed that none of the three psychological dimensions exerted a significant mediating effect between developmental feedback and learning engagement. Specifically, the indirect effect for autonomy was 0.346 (95% CI [−3.738, 4.450]); for competence, 0.364 (95% CI [−1.055, 2.202]); and for relatedness, 1.102 (95% CI [−1.705, 3.964]). Because all 95% confidence intervals included zero, and all *p*-values exceeded the 0.05 threshold (e.g., autonomy *p* = 0.864), the presence of mediation effects could not be substantiated (Table 7). Based on these results, there were no statistically significant differences in effect sizes among the respective mediation pathways.

**Table 7:** Results of bias-corrected bootstrap mediation analysis.

Indirect path	Effect	Boot SE	95% Boot CI	*p*	Conclusion
Group → Autonomy → Learning engagement	0.346	2.027	[−3.738, 4.450]	0.864	Not significant
Group → Competence → Learning engagement	0.364	0.820	[−1.055, 2.202]	0.657	Not significant
Group → Relatedness → Learning engagement	1.102	1.441	[−1.705, 3.964]	0.445	Not significant

Bootstrap resamples = 5,000. CI = confidence interval; SE = standard error.

In summary, while developmental feedback significantly enhanced international students’ needs for autonomy, competence, and relatedness (as demonstrated in RQ2), none of the three indirect pathways in the parallel mediation model achieved statistical significance (all 95% CIs included zero). This null finding warrants a dual interpretation through both methodological and theoretical lenses.

From a methodological perspective, the lack of significance may be attributed to limited statistical power, increasing the risk of a Type II error (false negative). Given the sample size (*N* = 53), simultaneously regressing three mediators likely inflated standard errors (SE), thereby reducing the model’s sensitivity to detect small-to-moderate indirect effects. Consequently, the quantitative model may have been insufficiently sensitive to capture the subtle psychological mechanisms underlying in explicit behaviors.

Theoretically, however, these results reveal a potential “theoretical disconnect” in classic SDT when applied to high-stakes, cross-cultural academic environments. Although psychological needs were successfully activated, they failed to translate into the behavioral engagement predicted by the theory. This decoupling of satisfaction and engagement suggests that the motivational transmission pathway—likened to an engine within a “black box”—was obstructed by contextual variables unique to cross-cultural adaptation.

The combination of methodological constraints and theoretical anomalies constitutes the most significant finding of the quantitative phase. Rather than merely reflecting model failure, it underscores the necessity of an explanatory sequential mixed-methods design. These results justify the subsequent qualitative phase, which will utilize triangulation to move beyond the limitations of the SDT framework, deconstruct the hidden barriers to motivational translation, and identify alternative mechanisms by which developmental feedback directly drives academic engagement.

### 5.5 Qualitative results: arousal mechanisms, mediational barriers, and alternative pathways (RQ4 + RQ5)

To address Research Questions 4 (the mechanisms of psychological need arousal) and 5 (the barriers to mediational pathways and the emergence of direct driving mechanisms), this study adopted a deviant case sampling strategy—a specialized form of purposive sampling.

Based on the standardized *Z*-scores of all key variables measured at Week 16 (W16), four theoretically representative categories of extreme cases were identified from the pool of 53 participants for semi-structured interviews (see Table 8). The objective of selecting these polar archetypes was to precisely deconstruct the nuanced process of psychological arousal and to elucidate the heterogeneous factors contributing to the “knowing–doing gap”—specifically, the disconnect between psychological satisfaction and actual behavioral engagement.

**Table 8:** Participant typologies for qualitative interviews.

Typology	Description	Rationale for selection
Type 1: Motivation-Awakened	High BPN satisfaction; High engagement	To validate the idealized SDT “motivation-to-behavior” path.
Type 2: Motivation-Engagement Decoupled	High BPN satisfaction; Low engagement	To identify translation barriers between cognition and action.
Type 3: Instruction-Driven Pragmatic	Low BPN growth; High engagement	To investigate the direct cognitive scaffolding effect (Path $c’$).
Type 4: Intervention-Resistant	Decline in all indicators	To identify boundary conditions and potential backlash effects.

#### 5.5.1 The catalytic and awakening mechanisms of basic psychological needs

The qualitative findings corroborated the significance of Path *a* in the quantitative model, affirming that developmental feedback serves as a potent catalyst for awakening students’ autonomy, competence, and relatedness.

Awakening autonomy: The negotiating discourse utilized by the instructor was pivotal. Interviewee S1 noted: *“The teacher frequently used a negotiating tone...this sense of being able to make my own decisions gave me a sense of control over the assignment.”* This empowerment dismantled traditional hierarchical dynamics, fostering a perceived locus of causality (Reeve, 2009).

Reconstructing competence: Feedback achieved a dimensional reduction by providing strategic support rather than mere correction. S1 described the process: *“The teacher did not just say ‘unclear logic,’ but drew a simple mind map... it was like building a ladder.”* This transformation of abstract critique into actionable steps allowed students to overcome frustration and experience mastery.

Bonding relatedness: Feedback functioned as emotional interaction rather than simple information transmission. S2 expressed: *“It not only closed the distance but dramatically enhanced trust... it felt like a team polishing a piece of work together.”* This continuous support constructed a psychological safe base essential for cross-cultural adaptation.

#### 5.5.2 Translation barriers: why psychological satisfaction failed to trigger engagement

The thematic analysis elucidated the non-significance of Path *b* by identifying three critical barriers that obstructed the translation of psychological energy into behavioral engagement.

Barrier 1: cross-cultural cognitive overload and resource competition. Even when motivated, students’ executive capacity was constrained by limited linguistic resources. S4 admitted: *“I had advanced ideas in my head... but my written expression ability could not support them, so eventually I just gave up revising.”* This discrepancy between conceptual motivation and linguistic execution, coupled with GPA trade-offs, created a severe cognitive bottleneck.

Barrier 2: the “comfort zone effect” induced by emotional compensation. High-quality feedback occasionally yielded unintended negative consequences: emotional satisfaction supplanted behavioral achievement. S3 remarked: *“The teacher already saw my attitude...this excellent relationship drained the sense of crisis that I absolutely had to submit a perfect assignment.”* When relatedness was “over-satisfied,” students exhibited behavioral inertia, feeling they had already achieved sufficient psychological validation.

Barrier 3: implicit transfer of engagement. Quantitative metrics may have failed to capture shifts in engagement modalities. Some participants (e.g., S4), while leaving fewer revision traces on academic documents, shifted their engagement to extracurricular cultural experiences (e.g., calligraphy clubs). This implicit engagement resulted in depressed scores within the standardized academic model.

Barrier 4: the authority–autonomy conflict under high power distance schemas. Qualitative analysis further reveals that beyond cognitive constraints, deep-seated sociocultural schemas constitute an implicit cultural barrier that obstructs the translation of autonomy into behavioral engagement. Traditional Chinese educational contexts are characterized by high Power Distance (Hofstede, 2011), wherein instructors are perceived as possessing inherent, unquestionable authority.

Interview findings suggest that these pre-existing “high-authority schemas” significantly distort how international students decode developmental feedback. Even when instructors deliberately employ egalitarian and negotiated discourse, some students—particularly those from low-power-distance cultural backgrounds—refract these “constructive suggestions” through the lens of cross-cultural stereotypes, misinterpreting them as non-negotiable mandates. As Participant S5 noted:

*“Even if the teacher says, ‘You might consider revising it this way,’ in a Chinese classroom setting, I feel it is a command that must be executed. I do not feel I have a choice.”*

This implicit cultural pressure effectively neutralizes or undermines the sense of autonomy that developmental feedback aims to foster. Consequently, while students may appear to be engaged in revisions, their behavior often stems from introjected regulation or compliance with authority rather than genuinely internalized autonomous will.

#### 5.5.3 Structured instrumental drive: the direct pathway

The interviews illuminated the strong significance of the direct effect (Path *c’*). Type 3 respondents (e.g., S6) indicated that their high engagement was not driven by intrinsic motivation but rather by the feedback functioning as a rigid checklist. The structured cognitive offloading allowed students to bypass the complex gestation phase of internal motivation, achieving immediate output through the mechanical execution of clear directives (Table 9).

**Table 9:** Qualitative coding scheme.

Level 3: Selective coding (Core Theme)	Level 2: Axial coding (Category)	Level 1: Open coding (Concept)	Representative quotes
Theme 1: Catalytic mechanism of psychological needs *(Corresponds to S1 and S2: Validating the first half of the quantitative model)*	1. Autonomy Support	1a. Empowerment through consultative discourse 1b. Breaking frames via flexible grading 1c. Establishing ownership of academic perspectives	“The teacher often uses a consultative tone… This feeling of being able to make my own decisions gives me a sense of control over my assignments.” (S1) “If you want to stick to this niche angle… the decision is yours.” (S2)
	2. Competence Reconstruction	2a. Reducing task complexity via cognitive scaffolding 2b. Addressing methodological blind spots 2c. Transforming frustration into a sense of mastery	“The teacher did not just say ‘unclear logic,’ but drew a simple mind map for me… It was like building a ladder.” (S1) “Instantly cleared my thoughts… I knew I could turn my ideas into high-quality output.” (S2)
	3. Relational Bonding	3a. Egalitarian dialogue 3b. Psychological safety zone via continuous interaction 3c. Non-evaluative emotional support	“Not only did we grow closer, but trust was greatly enhanced… It felt like a team polishing a work.” (S2) “I was careless with my homework that week, but the teacher still told me to get some rest… This sense of connection made me feel very safe.” (S1)
Theme 2: Translation barriers from motivation to behavior (Core Contribution) *(Corresponds to S3 and S4: Explaining non-significance in the latter half of the model)*	4. Resource Competition and Cognitive Overload	4a. Cross-cultural language burden 4b. Discrepancy between high cognitive expectations and low capabilities 4c. Zero-sum game between time and GPA	“I had advanced ideas in my head… but my written expression ability could not support them. In the end, I just gave up revising.” (S4) “The reading materials were too difficult! My brain could not process them… It wasn’t worth sacrificing my major course review for an elective.” (S3)
	5. Comfort Zone via Emotional Compensation	5a. Dissipation of crisis awareness and survival pressure 5b. Substituting behavioral achievement with psychological satisfaction	“The teacher had already seen my attitude… This good relationship removed the sense of crisis that I must submit a perfect assignment.” (S3) “I felt I had already ‘gained enough’… I was psychologically satisfied, so I slacked off in action.” (S4)
	6. Displacement of Learning Engagement Forms	6a. Stagnation of explicit assignment progress 6b. Increase in implicit cross-cultural interactions 6c. Extension into real-life experiences	“It did not translate into more characters in my document… but this semester I voluntarily signed up for Hanfu experiences and a calligraphy club. The time I spent on Chinese culture definitely increased!” (S4)
Theme3: Structured instrumental drive bypassing motivation *(Corresponds to S5 and S6: Discovering the direct effect path)*	7. Cognitive Offloading and Trigger	7a. Eliminating high-level internal cognitive friction 7b. Lowering activation energy for behavior 7c. Checklist effect	“It directly bypassed my painful stage of ‘self-doubt, struggling, and conceptualizing’ and became a clear checklist.” (S6) “I did not need to deal with highly draining questions like ‘where to start’… It directly triggered my execution.” (S5)
	8. Utility Asymmetry: Tool > Emotion	8a. Delayed/baseline nature of emotional support 8b. Immediate operability of instrumental scaffolding	“Emotional encouragement makes me feel the teacher is great, but I’m too tired now, I’ll study next week… However, looking at the specific task breakdown, I’d say ‘it only takes 5 min, I might as well fix it now.’” (S6) “Emotional support provides a baseline… but the instrumental utility of the specific steps is maximized.” (S5)
Theme 4: Backlash effects and boundaries of developmental feedback *(Corresponds to S7 and S8: Enhancing the critical depth of the study)*	9. Micromanagement and Autonomy Thwarting	9a. Feelings of being controlled induced by over-scaffolding 9b. Sense of negation regarding personal thought value 9c. Defensive resistance to intervention	“Nanny-style, dense annotations made me feel controlled… I became a typist for the teacher’s thoughts… generating strong feelings of resistance.” (S7)
	10. Emotional Exhaustion and Avoidance Coping	10a. Psychological overload from continuous developmental demands 10b. Negative visualization of constructive feedback 10c. Avoidance behavior to protect self-esteem	“After several consecutive weeks, my self-confidence was shattered… I did not even want to open that document to face the sense of oppression again.” (S8)

### 5.6 Synthesis of the explanatory sequential findings

**Figure 6:**
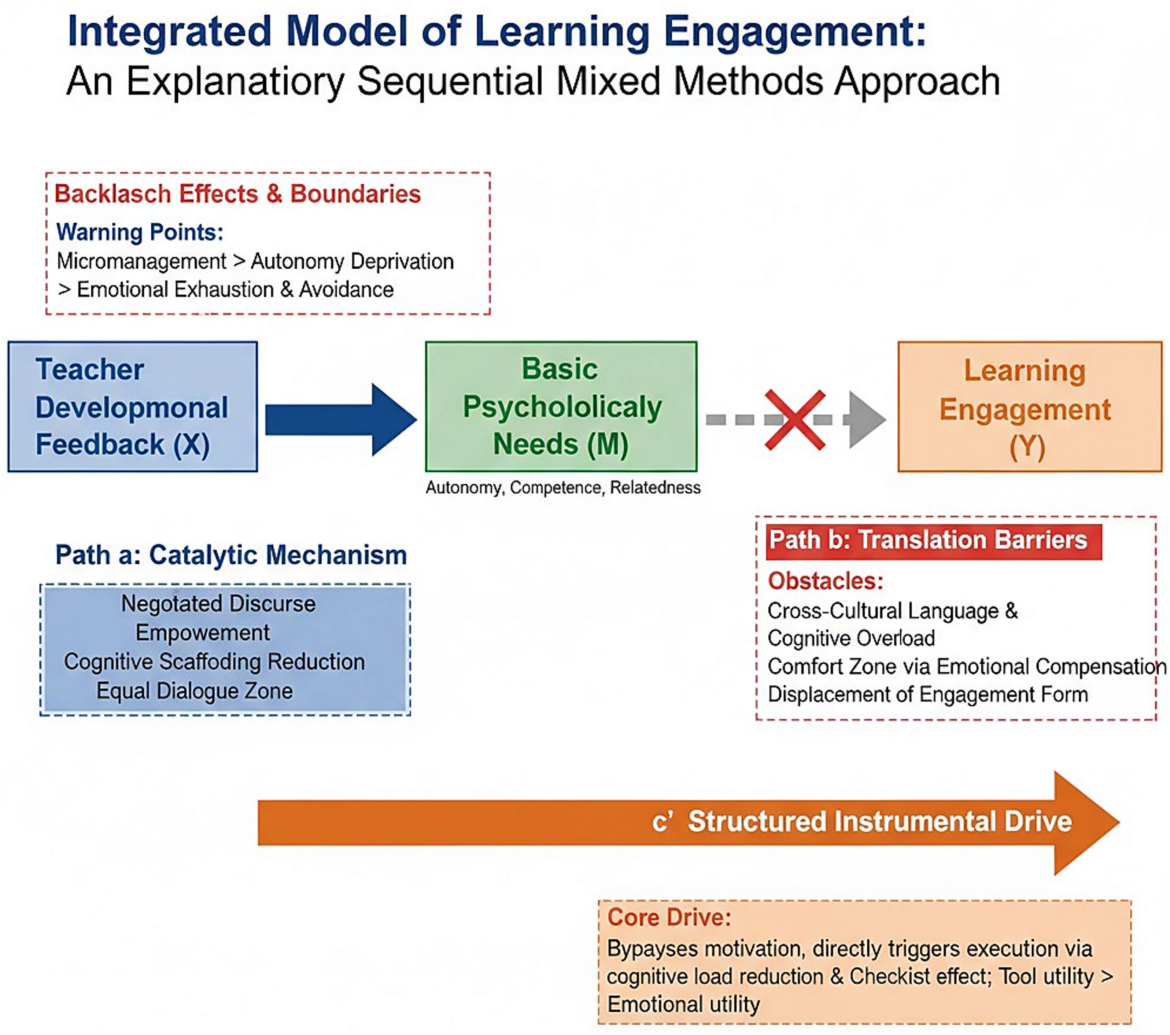
Integrated model of the effect of developmental feedback on learning engagement.

Integrating the quantitative and qualitative results (Figure 6), this study reaches three core conclusions regarding the instructional ecology of developmental feedback. (1) Quantitative Validation of Efficacy: Controlling for baseline levels, developmental feedback significantly outperforms traditional feedback in promoting learning engagement, *F* (1,50) = 340.179, *p* < 0.001. (2) Asymmetrical Awakening of Needs: Feedback successfully activates BPN via negotiating discourse and scaffolding. However, feeling satisfied does not automatically translate into generating engagement. The mediation model failed because high-level psychological satisfaction is a *necessary but insufficient* condition for behavioral change in cross-cultural settings. (3) The Dual-Path Mechanism: The Psychological Path, characterized by internalization is obstructed by cognitive overload, emotional comfort zones, and engagement transfer. In contrast, the Instrumental Path represents direct execution, wherein feedback acts as a checklist that drives student engagement by lowering the cognitive threshold for action.

In conclusion, while developmental feedback exerts a potent cognitive empowerment effect, educators must remain cognizant of the cross-cultural frictions between motivation and execution. An effective intervention must not only awaken internal needs but also provide the structured instrumental tools necessary to navigate entrenched linguistic and cognitive barriers.

## 6 Discussion

This study employed an explanatory sequential mixed-methods design to investigate the impact of developmental feedback on international students’ learning engagement and to delineate the underlying psychological mechanisms. At a macroscopic level, the results confirm the efficacy of developmental feedback in enhancing overall engagement. However, at a microscopic psychological level, the findings expose critical translation barriers inherent in the classic SDT model when applied to cross-cultural instructional contexts. Three core theoretical insights emerged: (1) basic psychological needs exhibited asymmetrical dynamic awakening trajectories, characterized by a U-shaped oscillation in competence and a delayed eruption of relatedness; (2) the satisfaction of these needs failed to significantly mediate learning engagement, primarily due to cross-cultural cognitive load and the emotional comfort zone effect; and (3) developmental feedback functioned essentially as a structured cognitive scaffold, directly driving behavioral engagement via a checklist effect.

### 6.1 The direct effect of developmental feedback on learning engagement

Consistent with the initial hypotheses, international students receiving developmental feedback demonstrated a significant enhancement in overall learning engagement compared to those receiving traditional evaluative feedback. This finding aligns with Hattie and Timperley’s (2007) feedback model and Shute’s (2008) meta-analysis on formative feedback. The congruence stems from the capacity of developmental feedback to not only identify the discrepancy between current and desired performance states but also to provide specific, actionable strategies for improvement. Viewed through the lens of Vygotsky’s Zone of Proximal Development (ZPD), interventions rich in cognitive scaffolding effectively lower the task activation threshold, thereby directly facilitating deeper cognitive and behavioral engagement (Van der Kleij et al., 2015).

Conversely, this finding diverges from Kluger and DeNisi’s (1996) classic meta-analysis, which cautioned that nearly one-third of feedback interventions actively diminish performance. This inconsistency can be attributed to the locus of the feedback. Traditional interventions frequently operate at the self-level, which can inadvertently trigger ego-defense mechanisms and subsequent ego-depletion. In contrast, the developmental feedback designed in this study was strictly directed at the task and process levels. By stripping away personal value judgments regarding the international students, the intervention mitigated the threats to self-esteem that are often amplified in cross-cultural learning environments, allowing the positive instructional effects to fully materialize.

### 6.2 Asymmetrical changes in psychological need dimensions

A critical finding of this study is that the awakening of the three basic psychological needs was not synchronous. Instead, they followed an asymmetrical dynamic trajectory characterized by an immediate activation of autonomy, a U-shaped oscillation of competence, and a late-stage eruption of relatedness.

These heterogeneous trajectories can be elucidated by Cognitive Load Theory (Sweller, 1988) and the U-Curve hypothesis of cross-cultural adaptation (Lysgaard, 1955). The initial decline in perceived competence suggests that high-quality developmental feedback, inherently accompanied by elevated academic standards, temporarily disrupts students’ pre-existing cognitive schemas. This disruption triggers short-term cognitive dissonance and a surge in intrinsic cognitive load (Festinger, 1957), a phenomenon corroborated by qualitative accounts from participants describing a transition from frustration to mastery. Furthermore, the delayed manifestation of relatedness supports the SDT tenet that genuine relationship-building requires sustained, non-evaluative interaction and the gradual accumulation of trust over time (Ryan and Deci, 2017).

While some empirical studies within the SDT framework posit that autonomy-supportive environments synchronously and rapidly elevate all three needs (Jang et al., 2010), the divergence observed here is likely attributable to the specific demographic characteristics of the sample. Previous research has predominantly focused on monocultural cohorts experiencing low cognitive strain. In contrast, the international students in the present study navigated the double burden of linguistic barriers and acculturative stress. These cross-cultural frictions create an inherent latency in the perception of competence and relatedness, thereby fracturing the presupposition of synchronous need satisfaction.

### 6.3 The nullification of the mediation pathway

Perhaps the most counterintuitive finding is that the satisfaction of the needs for autonomy, competence, and relatedness did not significantly mediate the relationship between developmental feedback and learning engagement, thereby challenging a core assumption of the SDT model in this specific context.

This knowing-doing gap finds robust theoretical support in Motivation-Volition Dual Model (Corno, 1993) and theories of Self-Regulated Learning (SRL). While the satisfaction of psychological needs addresses the motivation, the actual execution of learning engagement requires addressing the volition and regulatory strategies (Zimmerman, 2002). When confronting complex academic tasks, intrinsic motivation alone is often insufficient to overcome external interference and task difficulty; the translation of motivation into behavioral output heavily relies on volitional control.

This result contrasts with a substantial body of SDT literature (e.g., Niemiec and Ryan, 2009; Reeve, 2012), which posits that psychological need satisfaction acts as the bridge connecting environmental support to engagement. This discrepancy highlights two crucial contextual nuances: First, this study captured an emotional comfort zone effect—a nuance frequently overlooked in SDT applications. When the teacher-student relationship becomes highly supportive and emotionally satisfying, international students may lose their sense of academic urgency, resulting in motivational inertia. Second, by examining engagement within a context of high cross-cultural cognitive load, this study demonstrates that psychological comfort alone cannot directly catalyze academic output without the concurrent provision of actionable cognitive tools.

### 6.4 The dual-track framework: cross-cultural translation mechanisms and boundary effects

Drawing upon the proposed Motivation-Cognition Dual-Track framework, the qualitative deconstruction reconciles the apparent contradiction between the non-significant mediation pathways and the robust direct effects observed in the quantitative model. By integrating SDT with CLT, this study reveals the unique cross-cultural conflict boundaries that characterize the international student experience.

First, within this dual-track framework, cross-cultural barriers and emotional misalignment appear to sever the conventional “motivation-to-behavior” translation chain. On the Emotional Support Track (SDT perspective), the findings elucidate an “emotional comfort zone effect” that obstructs internalization: excessive relatedness satisfaction may inadvertently diminish students’ sense of academic urgency, thereby weakening their executive intent. More critically, on the CLT perspective, cross-cultural cognitive overload serves as the primary bottleneck. When the limited working memory of international students is exhausted by the heavy demands of linguistic and cultural decoding, even high levels of motivational satisfaction fail to translate into visible behavioral engagement.

Secondly, in the face of such cognitive overload, the significant direct effect of developmental feedback stems from the synergy of structured cognitive offloading and cultural authority compliance. In scenarios where the long-range motivational pathway is blocked, developmental feedback continues to drive engagement by functioning as a potent “structural scaffold,” significantly reducing extraneous cognitive load. However, the “task-list effect” identified in the qualitative analysis reflects a deeper authority compliance mechanism unique to the cross-cultural context (Kirschner et al., 2006). Under the “high power distance” (Hofstede, 2011) inherent in traditional Chinese pedagogical culture, international students often decode high-quality developmental scaffolding as “uncontestable instructions.” Consequently, they bypass the energy-intensive stage of autonomous internalization—as advocated by classic SDT—and adopt a pragmatic strategy of “authority-dependent execution.” This finding addresses the limitations of a singular motivational lens (SDT) by demonstrating that, in the early stages of cross-cultural adaptation, high-efficiency behavioral drivers may rely on a form of compliant compromise at the expense of autonomous exploration.

Finally, this high-authority feedback model operates within strict boundaries, beyond which perceived “over-scaffolding” triggers cultural conflict and need frustration. The “intervention resistance effect” observed in specific cases (e.g., Type 4) challenges the intuitive pedagogical consensus that “more detailed feedback is inherently superior” (Shute, 2008). When instructors engage in micromanagement through excessively dense annotations, the underlying perceived pedagogical authority clashes with the students’ burgeoning need for academic autonomy. In such instances, the cognitive scaffold is re-interpreted as a controlling mechanism that encroaches upon autonomous space. This phenomenon resonates with recent advancements in SDT regarding need frustration (Vansteenkiste et al., 2020), suggesting that excessive feedback can paradoxically induce defensive avoidance and emotional exhaustion. These results further echo the emphasis by Carless and Boud (2018) on the necessity of cultivating student feedback literacy. Without sensitivity to these cross-cultural psychological boundaries, even well-intentioned developmental interventions may cross the “authority-autonomy” red line, transforming the feedback from a catalyst for growth into a barrier to engagement.

### 6.5 Implications and future directions

The findings of this study not only extend the theoretical boundaries of cross-cultural motivation but also offer an actionable framework for educators to optimize feedback strategies within internationalized academic ecosystems. We propose three implementation standards for Cross-Cultural Developmental Feedback tailored to the specific needs of international students:

Standardizing Cognitive Offloading via Layered Scaffolding. To mitigate cross-cultural cognitive overload, instructors should move away from traditional, monolithic textual commentary. Instead, they should employ multimodal dimensionality reduction tools, such as logic-based mind maps or visual diagrams. Feedback should adhere to a hierarchical progression—prioritizing macro-level structural adjustments before micro-level linguistic corrections. By strictly controlling informational density in each feedback cycle, educators can prevent the exhaustion of students’ working memory capacity, ensuring that the feedback remains digestible and actionable.

Discourse Transformation: Negotiated Empowerment and Autonomy Support. To counteract the “High Power Distance” schemas prevalent in certain educational traditions and to avoid the psychological reactance triggered by “micromanagement,” instructors must consciously reconstruct their feedback discourse. This involves transitioning from imperative commands to hedged, open-ended negotiated discourse (e.g., “Have you considered exploring this perspective?” or “One potential alternative might be…”). More importantly, an institutional “right to dissent” should be established. By encouraging students to justify why they might choose *not* to adopt specific feedback, educators provide a formal mechanism that safeguards substantial academic autonomy and agency.

The Affective–Accountability Dual-Track Balance. Educators must move beyond the “attitude-as-engagement” myth—the misconception that high affective satisfaction automatically equates to high behavioral investment. While providing high levels of affective support (to satisfy relatedness), instructors must simultaneously implement transparent and non-negotiable academic rubrics. This “warm communication, firm standards” approach prevents an “affective compensatory trade-off,” where students might mistake emotional support for lowered academic expectations. By maintaining a dual-track focus on both empathy and accountability, educators ensure that motivation is successfully converted into rigorous academic output.

Furthermore, university administrators should adopt more diversified evaluation systems for international students. These systems should recognize and incentivize “implicit transfer”—the cognitive and emotional effort students invest in cultural adaptation and experiential learning—which is often overlooked in traditional academic performance metrics.

Future research should transcend traditional cross-sectional or sparsely repeated-measures designs by integrating methodologies such as Ecological Momentary Assessment (EMA). This would allow for the high-resolution, longitudinal tracking of psychological need oscillations immediately following feedback reception. Additionally, further investigation is warranted to map the precise threshold relationship between feedback density and the perceived locus of control, aiming to identify the optimal equilibrium of scaffolding that maximizes engagement without suppressing learner autonomy.

## 7 Conclusion

### 7.1 Theoretical and practical contributions

Employing an explanatory sequential mixed-methods design, this study explores the intervention effects and contextual mechanisms of developmental feedback on international student engagement. The central contribution lies in identifying the boundary conditions of classic SDT in cross-cultural academic settings and proposing a Motivation-Cognition Dual-Track Framework to reconcile motivational and cognitive perspectives.

First, while confirming the positive impact of developmental feedback on psychological needs, this study challenges the universalist assumption of SDT’s satisfaction-to-engagement mediation pathway within complex cross-cultural ecosystems. The findings reveal a significant decoupling between motivation and behavior when students confront severe linguistic and cultural barriers. This suggests that emotional satisfaction alone is insufficient to bridge the cognitive rupture inherent in heterogeneous cultural environments.

Secondly, the study elucidates that the efficacy of developmental feedback stems not only from motivational support but significantly from instrumental cognitive offloading. By functioning as a structural cognitive scaffold, high-quality feedback bypasses energy-intensive internalization stages and directly facilitates behavioral execution. This exploratory discovery provides empirical evidence for the boundary conditions of motivational theories and underscores the scientific necessity of integrating SDT with CLT in international education research.

To address the “authority-autonomy” conflict and the task-list effect identified in the qualitative analysis, this study proposes a three-pillar intervention framework to shift feedback from mere provision to effective uptake: (1) Structured Offloading: Instructors should transform abstract suggestions into “micro-scaffolds” (e.g., prioritized revision checklists or visual mind maps). By reducing extraneous cognitive load, these tools enable students with limited cognitive resources to initiate academic behaviors more efficiently. (2) Discursive Empowerment: In response to the high-authority nature of certain pedagogical traditions, teachers should employ “negotiated discourse” rather than “imperative annotations.” Framing feedback as a bidirectional dialogue can mitigate “need frustration” and safeguard students’ academic agency. (3) Dual-Track Accountability: A feedback-response closed-loop mechanism should be established. Beyond correcting errors, students should be guided to reflect on the underlying logic of the feedback. This approach facilitates a transition from passive execution to the proactive construction of feedback literacy, fostering long-term cross-cultural academic resilience.

### 7.2 Limitations

Despite its theoretical and practical insights, this study possesses several methodological and conceptual limitations that necessitate cautious interpretation of the findings.

First, there are inherent constraints regarding sample size and representativeness. The valid sample size is relatively small (*N* = 53) and was drawn from two natural classes at a single institution. The participants were predominantly from Asian countries. Consequently, the findings are deeply contextualized and may not be generalizable to the broader population of international students in China, particularly those with different academic majors, or diverse cultural backgrounds (e.g., students from Western cultures).

Second, the boundaries of causal inference must be acknowledged. Although this study matched baseline and pedagogical environment variables, the non-randomized quasi-experimental design means that unobserved systemic confounding variables—such as individual motivational traits or extrinsic academic support—cannot be entirely ruled out. Therefore, the statistical correlations derived from the quantitative models should be interpreted as exploratory evidence of directional trends rather than definitive causal conclusions.

Finally, the Motivation-Cognition Dual-Track mechanism identified in this study remains an exploratory hypothesis. As it was derived from a small-scale qualitative deconstruction and lacks validation through comparative theoretical models or confirmatory factor analysis (CFA), its universal validity and construct validity require further scrutiny. This mechanism is intended to provide empirical cues rather than established laws.

Future research is encouraged to conduct multi-center, multi-cultural randomized controlled trials (RCTs) with larger sample sizes to verify and extend the theoretical applicability of developmental feedback. Furthermore, pre-registered and replicable quantitative designs, incorporating SEM with explicit cognitive load measures, are needed to rigorously validate and refine the exploratory assertions proposed in this study.

### 7.3 Future directions: AI and feedback ecology

Looking ahead, the boundaries of educational feedback research are undergoing a profound paradigm shift. The mechanism of structured cognitive scaffolding identified in this study aligns seamlessly with the technological affordances of Generative Artificial Intelligence (GenAI). As GenAI becomes deeply embedded within classroom ecologies, future research must transcend the traditional binary teacher-student interaction framework, pivoting toward the exploration of complex, synergistic ecosystems, such as student-AI-teacher triads.

The highly effective mechanisms of instructional dimensional reduction and structured scaffolding found in human developmental feedback will provide the core algorithmic logic for developing AI pedagogical agents equipped with both high emotional intelligence and cognitive adaptability. Ultimately, the advancement of the field must not stagnate at exploring how the external environment should provide perfect feedback. Rather, it must direct its focus inward, dedicating efforts to cultivating learners’ digital feedback literacy. Empowering students to autonomously solicit, decode, and translate multi-source feedback within unpredictable cross-cultural and human-machine collaborative environments is imperative for realizing the fundamental leap from passive respondents to lifelong, self-regulated learners.

## Data Availability

The data presented in this study are available on request from the corresponding author.

## Appendix 1: semi-structured interview guide

The interview guide was theoretically grounded in Self-Determination Theory (SDT). The following questions served as a core framework, with the interviewer utilizing open-ended follow-up prompts based on the participants’ initial responses to elicit deeper reflections.

### Part I: context and nature of the feedback

1. Feedback Format: What kind of feedback did you receive from the instructor on your most recent assignment? Did it consist solely of a numerical grade, or did it also include specific comments?
2. Informational Value: After reviewing the instructor’s feedback, was it clear to you what specific steps you needed to take next?

### Part II: fulfillment of basic psychological needs (SDT framework)

1. Need for Competence: Did receiving this feedback alter your confidence in successfully completing this course? Are there any specific words or phrases in the feedback that made you feel capable or, conversely, inadequate?
2. Need for Autonomy: Did you perceive the instructor’s feedback as a direct mandate to make revisions, or as constructive suggestions offering you choices? When revising your assignment, did you feel compelled to do so out of obligation, or was it driven by a genuine desire to master the material?
3. Need for Relatedness: Upon receiving the feedback, how did you perceive the instructor’s underlying attitude toward you? Did this perception influence your overall view of the course?

### Part III: behavioral outcomes and engagement

1. Learning Engagement: In the days following the feedback, did you notice any changes in your study habits or overall engagement? For example, were your subsequent efforts driven by a desire to prove your abilities, or a wish to live up to the instructor’s expectations?

## References

[ref1] Andrade M. S. (2006). International students in English-speaking universities: adjustment factors. J. Res. Int. Educ. 5, 131–154. doi: 10.1177/1475240906065589

[ref2] Boud D. Molloy E. (2013). Rethinking models of feedback for learning: the challenge of design. Assess. Eval. High. Educ. 38, 698–712. doi: 10.1080/02602938.2012.691462

[ref3] Bureau J. S. Howard J. L. Chong J. X. Guay F. (2022). Pathways to student motivation: a meta-analysis of antecedents of autonomous and controlled motivations. Rev. Educ. Res. 92, 46–72. doi: 10.3102/00346543211042426, 35330866 PMC8935530

[ref4] Carless D. (2006). Differing perceptions in the feedback process. Stud. High. Educ. 31, 219–233. doi: 10.1080/03075070600572132

[ref5] Carless D. Boud D. (2018). The development of student feedback literacy: enabling uptake of feedback. Assess. Eval. High. Educ. 43, 1315–1325. doi: 10.1080/02602938.2018.1463354

[ref6] Carless D. Salter D. Yang M. Lam J. (2011). Developing sustainable feedback practices. Stud. High. Educ. 36, 395–407. doi: 10.1080/03075071003642449

[ref7] Carmichael C. Muir T. Callingham R. (2017). The impact of within-school autonomy on students' goal orientations and engagement with mathematics. Math. Educ. Res. J. 29, 219–236. doi: 10.1007/s13394-017-0200-z

[ref8] Comanaru R. Noels K. A. (2009). Self-determination, motivation, and the learning of Chinese as a heritage language. Can. Mod. Lang. Rev. 66, 131–158. doi: 10.3138/cmlr.66.1.131

[ref9] Corno L. (1993). The best-laid plans: modern conceptions of volition and educational research. Educ. Res. 22, 14–22. doi: 10.3102/0013189X022002014

[ref10] Creswell J. W. Plano Clark V. L. (2018). Designing and Conducting Mixed Methods Research. 3rd Edn. Thousand Oaks, CA: SAGE Publications.

[ref11] Csikszentmihalyi M. (1990). Flow: The Psychology of Optimal Experience. New York, NY: Harper & Row.

[ref12] Deci E. L. Ryan R. M. (2000). The "what" and "why" of goal pursuits: human needs and the self-determination of behavior. Psychol. Inq. 11, 227–268. doi: 10.1207/S15327965PLI1104_01

[ref13] Evans C. (2013). Making sense of assessment feedback in higher education. Rev. Educ. Res. 83, 70–120. doi: 10.3102/0034654312474350

[ref14] Faul F. Erdfelder E. Lang A.-G. Buchner A. (2007). G*power 3: a flexible statistical power analysis program for the social, behavioral, and biomedical sciences. Behav. Res. Methods 39, 175–191. doi: 10.3758/BF03193146, 17695343

[ref15] Fereday J. Muir-Cochrane E. (2006). Demonstrating rigor using thematic analysis: a hybrid approach of inductive and deductive coding and theme development. Int J Qual Methods 5, 80–92. doi: 10.1177/160940690600500107

[ref16] Festinger L. (1957). A Theory of Cognitive Dissonance. Stanford, CA: Stanford University Press.

[ref17] Fredricks J. A. Blumenfeld P. C. Paris A. H. (2004). School engagement: potential of the concept, state of the evidence. Rev. Educ. Res. 74, 59–109. doi: 10.3102/00346543074001059

[ref18] Gagné M. (2003). The role of autonomy support and autonomy orientation in prosocial behavior engagement. Motiv. Emot. 27, 199–223. doi: 10.1023/A:1025007614869

[ref19] Hattie J. Timperley H. (2007). The power of feedback. Rev. Educ. Res. 77, 81–112. doi: 10.3102/003465430298487

[ref20] Hayes A. F. (2018). Introduction to Mediation, Moderation, and Conditional process Analysis: A Regression-based Approach. 2nd Edn. New York, NY: Guilford Press.

[ref21] Henderson M. Ryan T. Boud D. Dawson P. Phillips M. Molloy E. . (2019). The usefulness of feedback. Act. Learn. High. Educ. 22, 229–243. doi: 10.1177/1469787419872393

[ref22] Hiver P. Al-Hoorie A. H. Vitta J. P. Wu J. (2021). Engagement in language learning: a systematic review of 20 years of research methods and definitions. Lang. Teach. Res. 25, 813–842. doi: 10.1177/13621688211001289

[ref23] Hofstede G. (2011). Dimensionalizing cultures: the Hofstede model in context. Online Read. Psychol. Cult. 2, 2307–0919.

[ref24] Hyland K. Hyland F. (2019). Feedback in second Language Writing: Contexts and Issues. 2nd Edn. Cambridge, United Kingdom: Cambridge University Press.

[ref25] Jang H. Reeve J. Deci E. L. (2010). Engaging students in learning activities: it is not autonomy support or structure but autonomy support and structure. J. Educ. Psychol. 102, 588–600. doi: 10.1037/a0019682

[ref26] Kahu E. R. (2013). Framing student engagement in higher education. Stud. High. Educ. 38, 758–773. doi: 10.1080/03075079.2011.598505

[ref27] Kahu E. R. Nelson K. (2018). Student engagement in the educational interface: understanding the mechanisms of student success. High. Educ. Res. Dev. 37, 58–71. doi: 10.1080/07294360.2017.1344197

[ref28] Kirschner P. A. Sweller J. Clark R. E. (2006). Why minimal guidance during instruction does not work: an analysis of the failure of constructivist, discovery, problem-based, experiential, and inquiry-based teaching. Educ. Psychol. 41, 75–86. doi: 10.1207/s15326985ep4102_1

[ref29] Kluger A. N. DeNisi A. (1996). The effects of feedback interventions on performance: a historical review, a meta-analysis, and a preliminary feedback intervention theory. Psychol. Bull. 119, 254–284. doi: 10.1037/0033-2909.119.2.254

[ref30] Koka A. Hein V. (2003). Perceptions of teacher's feedback and learning environment as predictors of intrinsic motivation in physical education. Psychol. Sport Exerc. 4, 333–346. doi: 10.1016/S1469-0292(02)00012-2

[ref31] Kuh G. D. (2001). Assessing what really matters to student learning inside the national survey of student engagement. Change 33, 10–17. doi: 10.1080/00091380109601795

[ref32] Kuh G. D. (2009). What student affairs professionals need to know about student engagement. J. Coll. Stud. Dev. 50, 683–706. doi: 10.1353/csd.0.0099

[ref33] Lincoln Y. S. Guba E. G. (1985). Naturalistic Inquiry. Thousand Oaks, CA: SAGE Publications.

[ref34] Lysgaard S. (1955). Adjustment in a foreign society: Norwegian Fulbright grantees visiting the United States. Int. Soc. Sci. Bull. 7, 45–51.

[ref35] Molloy E. Boud D. (2013). “Changing conceptions of feedback,” in Feedback in Higher and Professional Education, eds. Boud D. Molloy E. (London, United Kingdom: Routledge), 11–33.

[ref36] Narciss S. (2008). “Feedback strategies for interactive learning tasks,” in Handbook of research on educational communications and technology, 3rd Edn. eds. Spector J. M. Merrill M. D. Merriënboer J. van Driscoll M. P.. (New York, NY: Routledge) 125–144.

[ref37] Niemiec C. P. Ryan R. M. (2009). Autonomy, competence, and relatedness in the classroom: applying self-determination theory to educational practice. Theory Res. Educ. 7, 133–144. doi: 10.1177/1477878509104318

[ref38] Pascarella E. T. Terenzini P. T. (2005). How College Affects Students: A Third Decade of Research, vol. 2. San Francisco, CA: Jossey-Bass.

[ref001] Preacher K. J. Hayes A. F. (2008). Asymptotic and resampling strategies for assessing and comparing indirect effects in multiple mediator models. Behav. Res. Methods. 40, 879–891. doi: 10.3758/BRM.40.3.879 18697684

[ref39] Reeve J. (2009). Why teachers adopt a controlling motivating style toward students and how they can become more autonomy supportive. Educ. Psychol. 44, 159–175. doi: 10.1080/00461520903028990

[ref40] Reeve J. (2012). “A self-determination theory perspective on student engagement,” in Handbook of Research on Student Engagement, eds. Christenson S. L. Reschly A. L. Wylie C. (New York, NY: Springer), 149–172.

[ref41] Rienties B. Beausaert S. Grohnert T. Niemantsverdriet S. Kommers P. (2012). Understanding academic performance of international students: the role of ethnicity, academic and social integration. High. Educ. 63, 685–700. doi: 10.1007/s10734-011-9468-1

[ref42] Rienties B. Tempelaar D. (2013). The role of cultural dimensions of international and Dutch students on academic and social integration and academic performance in the Netherlands. Int. J. Intercult. Relat. 37, 188–201. doi: 10.1016/j.ijintrel.2012.11.004

[ref43] Ryan R. M. Deci E. L. (2017). Self-Determination Theory: Basic Psychological Needs in Motivation, Development, and Wellness. New York, NY: Guilford Press.

[ref44] Ryan R. M. Deci E. L. (2020). Intrinsic and extrinsic motivation from a self-determination theory perspective: definitions, theory, practices, and future directions. Contemp. Educ. Psychol. 61:101860. doi: 10.1016/j.cedpsych.2020.101860

[ref45] Ryan S. Reid L. (2016). "who is going to build the wall?" experiences of international students in the United States post-9/11. J. Int. Stud. 6, 467–482. doi: 10.32674/jis.v6i2.310

[ref46] Schaufeli W. B. Salanova M. González-Romá V. Bakker A. B. (2002). The measurement of engagement and burnout: a two sample confirmatory factor analytic approach. J. Happiness Stud. 3, 71–92. doi: 10.1023/A:1015630930326

[ref47] Shute V. J. (2008). Focus on formative feedback. Rev. Educ. Res. 78, 153–189. doi: 10.3102/0034654307313795

[ref48] Skinner E. A. Kindermann T. A. Furrer C. J. (2009). A motivational perspective on engagement and disaffection: conceptualization and assessment of children's behavioral and emotional participation in academic activities in the classroom. Educ. Psychol. Meas. 69, 493–525. doi: 10.1177/0013164408323233

[ref49] Skyrme G. (2007). Entering the university: the differentiated experience of two Chinese international students in a New Zealand university. Stud. High. Educ. 32, 357–372. doi: 10.1080/03075070701346915

[ref50] Steelman L. A. Levy P. E. Snell A. F. (2004). The feedback environment scale: construct definition, measurement, and validation. Educ. Psychol. Meas. 64, 165–184. doi: 10.1177/0013164403258440

[ref51] Sweller J. (1988). Cognitive load during problem solving: effects on learning. Cogn. Sci. 12, 257–285. doi: 10.1207/s15516709cog1202_4

[ref52] Tian L. Liu W. Gilman R. (2014). Basic psychological needs satisfaction, meaning in life, and depressive symptoms: a cross-lagged analysis. Soc. Indic. Res. 115, 857–874. doi: 10.1007/s11205-013-0254-6

[ref53] Tinto V. (2012). Completing College: Rethinking Institutional Action. Chicago, IL: University of Chicago Press.

[ref54] Valdés G. (2005). Bilingualism, heritage language learners, and SLA research: opportunities lost or seized? Mod. Lang. J. 89, 410–426. doi: 10.1111/j.1540-4781.2005.00314.x

[ref55] Van der Kleij F. M. Feskens R. C. Eggen T. J. (2015). Effects of feedback in a computer-based learning environment on students' learning outcomes: a meta-analysis. Rev. Educ. Res. 85, 475–511. doi: 10.3102/0034654314564881

[ref56] Vansteenkiste M. Ryan R. M. Soenens B. (2020). Basic psychological need theory: advancements, critical themes, and future directions. Motiv. Emot. 44, 1–31. doi: 10.1007/s11031-019-09818-1

[ref57] Winstone N. Carless D. (2020). Designing Effective Feedback Processes in Higher Education: A Learning-Focused Approach. London, United Kingdom: Routledge.

[ref58] Winstone N. E. Nash R. A. Parker M. Rowntree J. (2017). Supporting learners’ agentic engagement with feedback: a systematic review and a taxonomy of recipience processes. Educ. Psychol. 52, 17–37.

[ref59] Zepke N. Leach L. (2010). Improving student engagement: ten proposals for action. Act. Learn. High. Educ. 11, 167–177. doi: 10.1177/1469787410379680

[ref60] Zhao C.-M. Kuh G. D. Carini R. M. (2005). A comparison of international student and American student engagement in effective educational practices. J. High. Educ. 76, 209–231. doi: 10.1353/jhe.2005.0018

[ref61] Zhou J. (2003). When the presence of creative coworkers is related to creativity: role of supervisor close monitoring, developmental feedback, and creative personality. J. Appl. Psychol. 88, 413–422. doi: 10.1037/0021-9010.88.3.413 12814291

[ref62] Zimmerman B. J. (2002). Becoming a self-regulated learner: an overview. Theory Pract. 41, 64–70. doi: 10.1207/s15430421tip4102_2

